# SIRT1 restores mitochondrial structure and function in rats by activating SIRT3 after cerebral ischemia/reperfusion injury

**DOI:** 10.1007/s10565-024-09869-2

**Published:** 2024-05-20

**Authors:** Manli Chen, Ji Liu, Wenwen Wu, Ting Guo, Jinjin Yuan, Zhiyun Wu, Zhijian Zheng, Zijun Zhao, Qiang Lin, Nan Liu, Hongbin Chen

**Affiliations:** 1 Department of Neurology, Fujian Medical University Union Hospital, Fuzhou, China; 2 Department of Rehabilitation, Fujian Medical University Union Hospital, Fuzhou, China; 3 Fujian Key Laboratory of Molecular Neurology, Fujian Medical University, Fuzhou, China; 4 Institute of Clinical Neurology, Fujian Medical University, Fuzhou, China

**Keywords:** SIRT1, SIRT3, Cerebral ischemia/reperfusion, Oxidative stress, Neuronal apoptosis, Mitochondrial structure and function

## Abstract

Mitochondrial dysfunction contributes to cerebral ischemia–reperfusion (CI/R) injury, which can be ameliorated by Sirtuin-3 (SIRT3). Under stress conditions, the SIRT3-promoted mitochondrial functional recovery depends on both its activity and expression. However, the approach to enhance SIRT3 activity after CI/R injury remains unelucidated. In this study, Sprague–Dawley (SD) rats were intracranially injected with either adeno-associated viral Sirtuin-1 (AAV-SIRT1) or AAV-sh_SIRT1 before undergoing transient middle cerebral artery occlusion (tMCAO). Primary cortical neurons were cultured and transfected with lentiviral SIRT1 (LV-SIRT1) and LV-sh_SIRT1 respectively before oxygen–glucose deprivation/reoxygenation (OGD/R). Afterwards, rats and neurons were respectively treated with a selective SIRT3 inhibitor, 3-(1H-1,2,3-triazol-4-yl) pyridine (3-TYP). The expression, function, and related mechanism of SIRT1 were investigated by Western Blot, flow cytometry, immunofluorescence staining, etc. After CI/R injury, SIRT1 expression decreased in vivo and in vitro. The simulation and immune-analyses reported strong interaction between SIRT1 and SIRT3 in the cerebral mitochondria before and after CI/R. SIRT1 overexpression enhanced SIRT3 activity by increasing the deacetylation of SIRT3, which ameliorated CI/R-induced cerebral infarction, neuronal apoptosis, oxidative stress, neurological and motor dysfunction, and mitochondrial respiratory chain dysfunction, promoted mitochondrial biogenesis, and retained mitochondrial integrity and mitochondrial morphology. Meanwhile, SIRT1 overexpression alleviated OGD/R-induced neuronal death and mitochondrial bioenergetic deficits. These effects were reversed by AAV-sh_SIRT1 and the neuroprotective effects of SIRT1 were partially offset by 3-TYP. These results suggest that SIRT1 restores the structure and function of mitochondria by activating SIRT3, offering neuroprotection against CI/R injury, which signifies a potential approach for the clinical management of cerebral ischemia.

**Graphical Abstract:**

1. SIRT1 is downregulated after cerebral ischemia/reperfusion injury.

2. SIRT1 can increase the deacetylation of SIRT3 and enhance the activity of SIRT3 after cerebral ischemia/reperfusion injury.

3. SIRT1 enhances the mitochondrial structure repair and functional recovery by activating SIRT3 after cerebral ischemia/reperfusion injury in rats, thereby promoting neurological function.

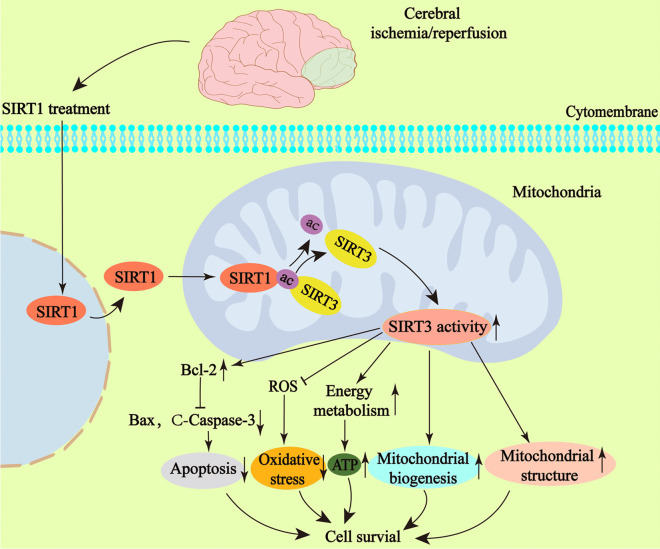

**Supplementary Information:**

The online version contains supplementary material available at 10.1007/s10565-024-09869-2.

## Introduction

Ischemic stroke is a most common refractory encephalopathy that may exert crippling effects on human health (Li et al. 2019; Li et al. 2020a, b, c, d). Currently, vascular recanalization is considered as the best treatment for ischemic stroke, but its efficacy has largely been compromised due to the narrow time window for treatment and reperfusion-induced secondary injuries (Nogueira et al. 2018; Thomalla et al. 2018). Accordingly, an exploration of the related mechanisms underlying the cerebral ischemia–reperfusion (CI/R)-induced injury is pivotal for seeking optimal alternatives to mitigate the CI/R-induced pathophysiological injury.

Intracellularly, mitochondria play a vital role in oxidative phosphorylation, electron transportation and energy metabolism. After CI/R injury, mitochondria produce an excess of reactive oxygen species (ROS), leading to potential oxidative harm to the mitochondria (Orellana-Urzúa et al. 2020; Shi et al. 2021). This process may pry open the opening of the mitochondrial permeability transition pore (mPTP), triggering a decline in mitochondrial membrane potential (ΔΨm) and the release of cytochrome c (Cyt c) from the mitochondria (Chen et al. 2022). Ultimately, the whole process activates the related caspases, inducing cell death (Huang et al. 2016). Several studies have demonstrated that neurodegenerative diseases can result in a decrease in mitochondrial DNA (mtDNA) and mitochondrial biogenesis (Li et al. 2017a, b) and that reduced blood supply can disrupt the energy balance, respiratory chain, and adenosine triphosphate (ATP) synthesis (Geldon et al. 2021; Stefanatos and Sanz 2018). Therefore, it is crucial to restore the mitochondrial structure and function after CI/R injury.

Molecularly, as a NAD^+^-dependent histone deacetylase, Sirtuin-3 (SIRT3) is mainly expressed in mitochondria (Salvatori et al. 2017; Wu et al. 2019). Available literature evidences that SIRT3 can regulate the mitochondrial function and alleviate mitochondrial oxidative damage in cardiovascular diseases (W Sun et al. 2018a, b), osteoarthritis (He et al. 2020), intestinal ischemia–reperfusion (I/R) injury (Z Wang et al. 2020), CI/R injury (Tu et al. 2019; Xie et al. 2018). Previous research has documented that under stress conditions, the SIRT3-promoted recovery of mitochondrial function is related not only to the expression of SIRT3 but also to its activity (Koentges et al. 2019; Kwon et al. 2017; Yi et al. 2019). Still other studies have evidenced that an upregulated SIRT3 activity can attenuate the oxidative stress and apoptosis in cardiac cells after myocardial I/R injury (Zhai et al. 2017). Furthermore, the activity of SIRT3 may be modulated by the acetylation/deacetylation of SIRT3 (Kwon et al. 2017; Yi et al. 2019), in which an enhanced acetylation of SIRT3 indicates a lower activity. However, little research has illuminated an optimal means to enhance the deacetylation of SIRT3 so as to augment the activity of SIRT3 after the CI/R injury.

Sirtuin-1 (SIRT1), a type of histone deacetylase enzyme that relies on NAD^+^ for its activity, can deacetylate histone and nonhistone proteins (Gurd 2011; Li et al. 2020a, b, c, d), which is mainly located in nucleus and can appear in the cytoplasm in certain conditions (Hernández-Jiménez et al. 2013; Jin et al. 2007). An earlier research has reported that SIRT1 overexpression can preserve SIRT3 activity in human pulmonary arteriolar smooth muscle cells (PASMCs) after the H/R exposure (hypoxia/reoxygenation) (P Li et al. 2017a, b). However, it remains unelucidated whether SIRT1 can promote SIRT3 activity after CI/R injury.

Respectively employing transient models of middle cerebral artery occlusion (tMCAO) and oxygen and glucose deprivation/reoxygenation (OGD/R), the present study explored the role of SIRT1 in protecting mitochondrial structure and function following CI/R injury and the underlying mechanisms. We found that after CI/R injury, SIRT1 expression decreased in vivo and in vitro, and that SIRT1 overexpression enhanced the deacetylation and activity of SIRT3, restoring the mitochondrial structure and function. These findings signify that SIRT1 could be a potential focus in managing cerebral ischemia.

## Materials and methods

### Animals

Sprague–Dawley (SD) rats (aged 16-18 weeks and weighed 250–280 g, male) were procured from Experimental Animal Center of Fujian Medical University. The rats had unrestricted access to both water and food and were maintained in a room (temperature: 22 ± 1 ℃; humidity: 50–60%) on a 12-h light/dark cycle.

### Surgical procedure

As described previously, the CI/R model was constructed via transient middle cerebral artery occlusion (tMCAO) (Sun et al. 2018a, b). In brief, the animals underwent an intraperitoneal injection of pentobarbital sodium (40 mg/kg). Afterwards, the neck was disinfected with iodophor before making a 1.5 cm incision with surgical scissors to expose the left common carotid artery (CCA), internal carotid artery (ICA) and external carotid artery (ECA). Next, CCA and ECA were occluded using temporary atraumatic clips. A silicon-coated nylon monofilament (0.2 mm in diameter) (Doccol, Corporation, USA) was carefully inserted into the ICA (Insertion length:18-20 mm). The silicon-coated nylon monofilament was gently withdrawn 90 min after the tMCAO procedure to ascertain reperfusion.

### Adeno-associated virus injection

As previously described, AAV–Empty, AAV-SIRT1 or AAV-sh_SIRT1 (at a dose of 1.04 × 10^10^gc; Hanbio, Shanghai, China) was respectively injected into the cortex and ipsilateral striatum of rats 21 days before tMCAO surgery (Zheng et al. 2018). In brief, a 10 ul volume micro-syringe (Hamilton, Reno, NV) was injected into the bregma origin of the coordinate system: mediolateral [−]2.5 mm, anterior-posterior 0.2 mm. The depth of insertion was 2.5 mm and 4.5 mm. The syringe remained in situ for 3 min after each injection and 15 min after the final injection to prevent any reflux after the injection.

### Cultured primary rat cortical neurons and oxygen–glucose deprivation/reoxygenation (OGD/R)

As described previously, with minor modifications, the OGD/R model was established (Yi et al. 2019). Briefly, the cerebral cortex from SD rat embryos (aged 16–18 days) was dissected. The cerebral cortex was then placed in PBS on an ice plate after the removal of pia mater and blood vessels with ophthalmic forceps. Subsequently, the cerebral cortex was sliced into particles (1mm^3^ in size), gently blown, and resuspended with 2 mL of PBS. Next, the resuspended particles were transferred into a 15 mL centrifuge tube with 0.25% trypsin (Cat# 25200–072, Gibco, NY, USA) and placed in a cell incubator (37 °C) for digestion for 30 min. Then, the cortical neurons were isolated and centrifuged before the removal of the supernatant. The cortical neurons subsequently received a 10 mL neuronal medium (containing 96% Neurobasal, 1% 50 U/ml penicillin, 2% B27, 1% 0.5 mM glutamine) (Ca# 21103–049, Cat# 17504–044, Cat# 15140–122, Cat# 35050–061, respectively; all from Gibco, NY, USA). Scattered cells were cultured in coverslips (24 × 24 mm^2^) and plates. After a PBS wash, the cells were refreshed with the same medium and further cultured in an incubator with 5% CO_2_.

After 7 days of plating, the cortical neurons underwent 3 PBS washes and were subsequently exposed to a DMEM that did not contain glucose (Cat# 11966–025; Gibco, NY, USA) before being cultivated in an anaerobic chamber for 120 min. Afterwards, the cortical neurons were returned to their initial culture environment for 12 h.

### Lentivirus transfection

As per the instructions from the manufacturer, the cortical neurons were transfected with 3 MOI of puromycin-resistant or GFP-contained LV-vector, LV-SIRT1 or LV-sh_SIRT1, respectively (Hanbio, Shanghai, China). Three days after the transfection, the cortical neurons underwent diverse treatments and were then subjected to distinct experimental procedures. Neurons transfected with puromycin-resistant lentiviruses were utilized to determine ROS levels and underwent flow cytometric analysis, whereas those transfected with GFP-containing lentiviruses were subjected to the other experimental assessments.

### Chemical administration

3-(1H-1,2,3-triazol-4-yl) pyridine (3-TYP, HY-1083; MedChemExpress, USA) was dissolved in dimethyl sulfoxide (DMSO, less than 2%). The animals received an intraperitoneal injection of 3-TYP at a dosage of 50 mg/kg every other day for six consecutive days prior to the tMCAO surgery (Zhai et al. 2017). Neurons were treated with 3-TYP (5uM) for 4 h prior to the OGD/R exposure (C Wang et al. 2019; Wu et al. 2020).

### Neurobehavioral tests

The modified neurological severity score (mNSS) was performed to evaluate the sensory, reflex, balance, and motor behaviors of the animals. A scale of 0 to 18 was employed to score the neurological function, where a score of 0 denoted no neurological impairment and a score of 18 represented the severest neurological deficit (N Liu et al. 2011).

As previously described, a rat rotarod 47700 tester was employed to assess the motor coordination of rats (Ugo Basile, Milan, Italy) (Shiotsuki et al. 2010). Before the experiment, the rats were trained to stay on the rotating rod as it increased progressively from 4 to 40 rpm within a span of 5 min. The trial test was repeated 10 times and the durations of rats staying on the rod were averaged and recorded.

As previously described, a YLS-13A grip strength tester was adopted to evaluate the rats’ forelimb grip strength (Yiyan Technology, Shang Dong, China) (Xiao et al. 2020). The rats were placed on a flat plate and allowed to grasp the sensing rod. They were pulled by the tail until their bodies were parallelly lifted off the plate. The experimenter pulled the rat slowly backwards until the animal released the sensing rod. The experiment was replicated 10 times and the grip strength was averaged and recorded.

### Magnetic resonance imaging (MRI)

As previously described, rats underwent MRI at 72 h after CI/R injury (7-T, Bruker Medizintechnik, Germany) (Harada et al. 2007). Briefly, the animals underwent inhalational anesthesia via an exposure to a blend of 1.5–2% isoflurane and oxygen for 5 min. The respiratory status of the rats was assessed using a respiratory detection system, with the respiratory rate maintained at 25–30 breaths/min. T2-weighted images were acquired with the following sequence (TubroRARE): Slice thickness (ST) = 0.56 mm, Slices = 48, Field of view (FOV) = 35 × 35mm^2^, Time of repetition (TR) = 5200 ms, Matrix = 256 × 256, Time of echo (TE) = 32 ms, Total scan time = 11 min 5 s 600 ms. The images were analyzed using Image J software. The infarct volume was determined by the equation: The percentage of cerebral infarct volume = infarct area volume/brain volume × 100%.

### Real-time quantitative PCR (RT-qPCR)

The RNA extraction was carried out with a Trizol Up Plus RNA Kit (Cat# ER501-01; TransGen, Beijing, China) to obtain total RNA from peri-ischemic cortex tissues. Then, mRNA was converted to cDNA with a 1st Strand cDNA Synthesis Kit (Cat# 11121ES60; YEASEN, Shanghai, China). Finally, RT-qPCR was conducted utilizing a Prism 7500 thermal cycler (Applied Biosystems, USA) and an RT-qPCR SYBR Green Kit (Cat# 11143ES50, YEASEN, Shanghai, China). The primer details are summarized in Table S1. The levels of mRNA were adjusted against GAPDH mRNA levels and determined using the 2^−ΔΔCT^ formula.

### Western blotting

As described previously, Western Blot was conducted (Karda et al. 2017; Xie et al. 2022). In brief, proteins (20–40 ug) were subjected to electrophoresis, electroporation, blocking, and incubation with the primary antibodies and the corresponding secondary antibodies (Table S2). The chemiluminescence system (Bio-Rad, CA, USA) was adopted to visualize the target proteins. Finally, the protein levels were analyzed with the ImageJ software and then standardized against Lamin B1, β-actin, or Tomm20 on an individual basis.

### The isolation of mitochondria, cytoplasm, and nucleus

Mitochondria, cytoplasm, and nucleus were isolated using a Mitochondrial Fractionation Kit (Cat# 40015; Active Motif, Shanghai, China) following the protocol from the manufacturer. Briefly, the neurons were spun at 600 g for 5 min at 4 ℃. After the supernatant removal, the cell pellet was resuspended in 5 ml PBS and spun at 600 g for 5 min at 4 ℃ before another supernatant removal. Afterwards, the cell pellet was homogenized using 30–50 strokes with a homogenizer and spun at 800 g for 20 min at 4 ℃. The resulting pellet was the nucleus. Finally, the supernatant was spun at 10,000 g for 20 min at 4 ℃, producing the mitochondria and the cytosolic fraction. The protein concentration of each fraction was determined against a BSA standard curve by a Bio-Rad assay.

### Measurement of mitochondrial bioenergetics

As per the instructions from the manufacturer, mitochondrial oxygen consumption rate (OCR) was determined with a Seahorse XF Cell Mito Stress Test Kit (Cat# 103,015–100, Seahorse Bioscience, Copenhagen, Denmark) (Paliwal et al. 2018). The cartridge was calibrated with purified water before OCR determination. Then, the mitochondria-containing plate was inserted into the analyzer and successively received oligomycin at 5 µmol/L, FCCP at 5 µmol/L, and rotenone/antimycin A at 0.5 µm to identify key assessment parameters.

### Measurement of MDA, ATP content, SOD and GSH-Px

The level of malondialdehyde (MDA), ATP content, the activities of superoxide dismutase (SOD) and glutathione peroxidase (GSH-Px) were respectively detected with a Lipid Peroxidation MDA Assay Kit, an enhanced ATP assay kit, SOD Assay Kit (Cat# S0131S; Cat# S0027; Cat# S0103; all from Beyotime, Shanghai, China), GSH-Px Assay Kit (Cat# A005, Nanjing Jiancheng Bioengineering Institute, Nanjing, China) as per the protocol from the manufacturer.

### Immunofluorescent staining

As previously described, the immunofluorescence was performed with minor modification (Q Y Zhang et al. 2019). Briefly, the tissue sections were blocked before an overnight incubation with primary antibodies (Table S4). Next, they were subjected to a 2-h incubation with the appropriate secondary antibody (Table S4). DAPI (Cat# C3606, Beyotime, Shanghai, China) was employed to stain the cell nuclei for 15 min. Lastly, the tissue sections were visualized by confocal microscopy (LSM 750, Zeiss, Gottingen, Germany).

### Detection of mitochondrial respiratory chain

As per the instructions from the manufacturer, the levels of mitochondrial complexes I − V in the cerebral mitochondria were detected with the biochemical analysis kits (Cat# BC0515; Cat# BC3235; Cat# BC3245; Cat# BC0945; Cat# BC1445; all from Solarbio, Beijing, China). The levels of mitochondrial complexes I–V were expressed in nmol/min of the cerebral mitochondrial protein.

### Molecular dynamic (MD) simulation procedures

The protein structures of SIRT3 (https://www.uniprot.org/uniprotkb/ B2RZ31/entry) and SIRT1 (https://www.uniprot.org/Uniprotkb/A0A0G2JZ79/entry) were downloaded from Uniprot. The protein structures were modeled with AlphaFold2 software (Jumper et al. 2021), visualized, and depicted with Pymol software (Schrödinge, Inc.). The results were analyzed with LigPlot + software package (Laskowski and Swindells 2011).

MD simulations were performed with Gromacs 2019.6 using the amber14sb force field (Van Der Spoel et al. 2005). Subsequently, MD simulation was performed at normal temperature and pressure for 500 ns. The value of root-mean-square deviation (RMSD) represented the stability of backbone structures in the complex. Radius of gyration (Rg) was adopted to estimate the proximity of the architecture. The solvent accessible surface area (SASA) indicated the solvent-accessible surface areas of the compounds. The hydrogen bond number (HBnum) was employed to describe the hydrogen bond contact between two proteins. The algorithm was realized by Gmx_MMPBSA (Valdes-Tresanco et al. 2021).

### Statistical analysis

Numeric statistics from animal and cell experiments were presented as the mean ± standard error of the mean (SEM) and processed with SPSS 20.0 software. Data normality was examined by the Shapiro–Wilk normality test and variance homogeneity was assessed by Levene's test. Data with equal variance were analyzed by one-way ANOVA with Bonferroni's post hoc test; those with unequal variance by one-way ANOVA with Dunnett's T3 post hoc test; and those collected from the same subjects at different time points by two-way repeated-measures ANOVA with Bonferroni post hoc test. The statistical significance was defined as follows: *p < 0.05, **p < 0.01, ***p < 0.001 as compared with the Sham or Control group; ^#^*p* < 0.05, ^##^*p* < 0.01, ^###^*p* < 0.001 as compared with the tMCAO + AAV-Empty or OGD/R + LV-Vector group; ^$^*p* < 0.05, ^$$^*p* < 0.01, ^$$$^*p* < 0.001 as compared with the tMCAO or OGD/R group; ^&^*p* < 0.05, ^&&^*p* < 0.01, ^&&&^*p* < 0.001 as compared with the tMCAO + AAV-SIRT1 or OGD/R + LV-SIRT1 group.

## Results

### SIRT1 interacts with SIRT3 before and after CI/R

**Fig. 1:**
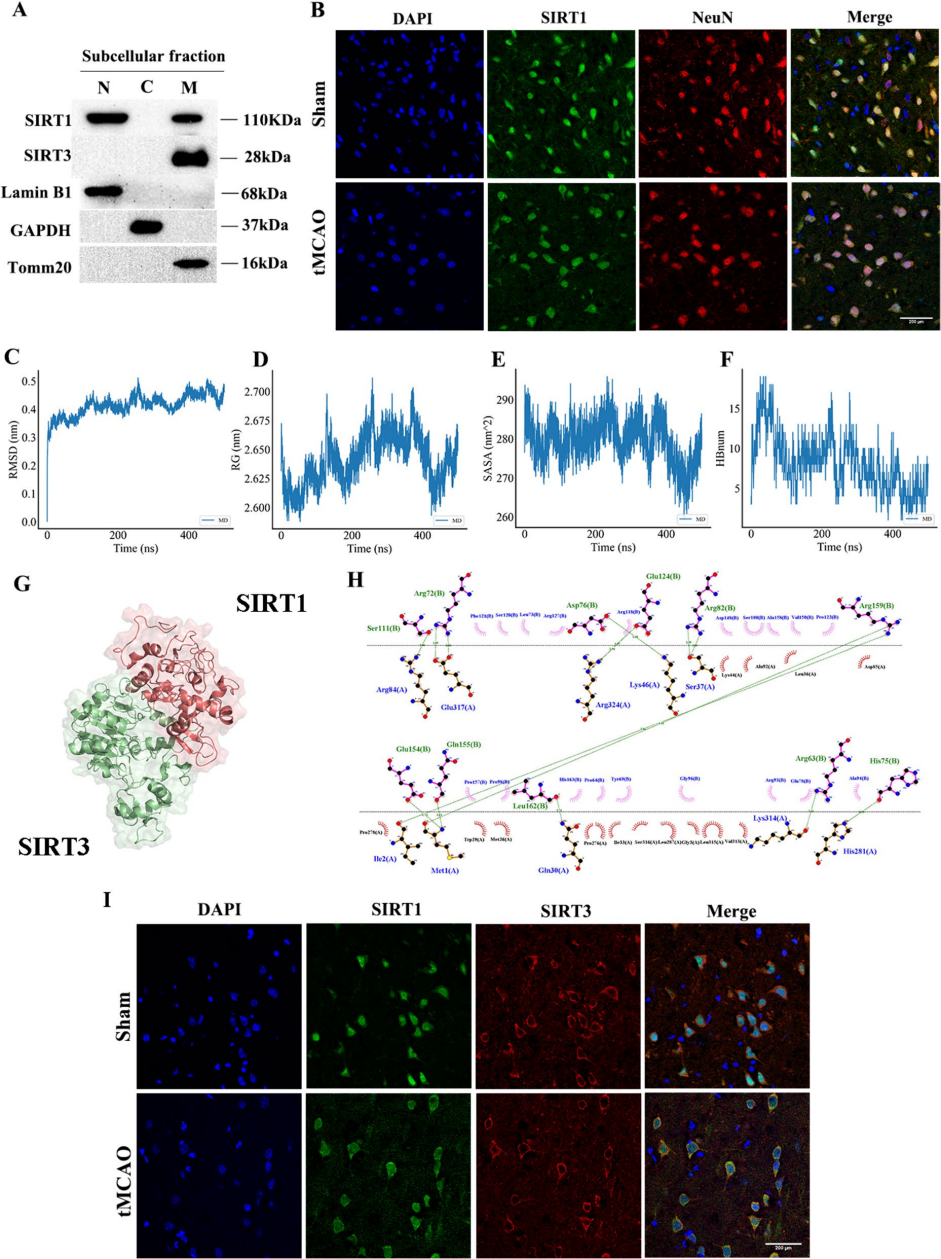
The interaction between SIRT1 and SIRT3 before and after CI/R. **A** Representative immunoblots of SIRT1 and SIRT3 in each subcellular fractionation in neurons. *n* = 3. **B** Representative confocal images of co-localization of SIRT1 and NeuN (neuron marker) (63 × objective). *n* = 3. Scale bar: 200 μm. **C** RMSD. **D** Rg. **E** SASA. **F** HBnum. **G** SIRT1 bound to SIRT3 in a 3D space. **H** 2D ligand-residue analysis. **I** Representative confocal images of co-localization of SIRT1 and SIRT3 (63 × objective). *n* = 3. Scale bar: 200 μm

Western Blotting of cortical neurons and immunofluorescence analysis of the cortex were performed to confirm the subcellular expression of SIRT1 and SIRT3. The former indicated that SIRT1 was located in both nucleus and mitochondria and SIRT3 was only expressed in mitochondria (Fig. 1A). The latter revealed that SIRT1 was co-localized with NeuN (a neuronal marker) and was mainly localized in the nucleus in the Sham group, which was partly transferred to the cytoplasm in the tMCAO group (Fig. 1B).

Subsequently, we conducted a molecular dynamic (MD) simulation to explore the interaction between SIRT1 and SIRT3. The values of RMSD, Rg, SASA and HBnum were computed according to the trajectory data. RMSD reported three obvious rising periods in the system, namely, 0–100, 150–280 and 400–450 equilibria, with the final equilibrium leveling off at 0.4 nm (Fig. 1C). Rg showed three peak changes before entering the final equilibrium (Fig. 1D). SASA indicated a decrease in 0–100 ns, an increase in 100–220 ns, and a fluctuated decrease until a minimum in 420 ns, which was consistent with the trends in RMSD and Rg (Fig. 1E). The change curve of HBnum showed an initial increase in the contact of the system and a subsequent decrease with fluctuation for 5–15 times (Fig. 1F). The analysis of free energy revealed that the SIRT1-SIRT3 complex had a binding affinity score of -45.47 kcal/mol (Table S2), indicating a successful binding of SIRT1 to SIRT3. The diagram of 3D and 2D ligand interaction illustrated the binding affinity of the SIRT1-SIRT3 complex. The hydrogen bonds between them were as follows: Ser111(B)-Arg84(A), Arg72(B)-Glu317(A), Asp76(B)-Lys46(A), Glu124(B)-Arg324(A), Arg82(B)-Ser37(A), Arg159(B)-Ile2(A)/Met1(A), Glu154(B)-Met1(A), Gln155(B)-Met1(A), Leu162(B)-Gln30(A), Arg63(B)-Lys314(A), and His75(B)-His281(A) (Fig. 1G-H). The findings indicate that the binding affinity between SIRT1 and SIRT3 is largely influenced by hydrogen bonding and hydrophobic interactions.

We further verified the co-localization of SIRT1 and SIRT3 in the cortex by immunofluorescence analysis (Fig. 1I). Taken together, the above results evidence an intense interaction between SIRT1 and SIRT3.

### The effect of SIRT1 on the neurological function, infarct volume, and the acetylation and activity of SIRT3 in rats after CI/R

**Fig. 2:**
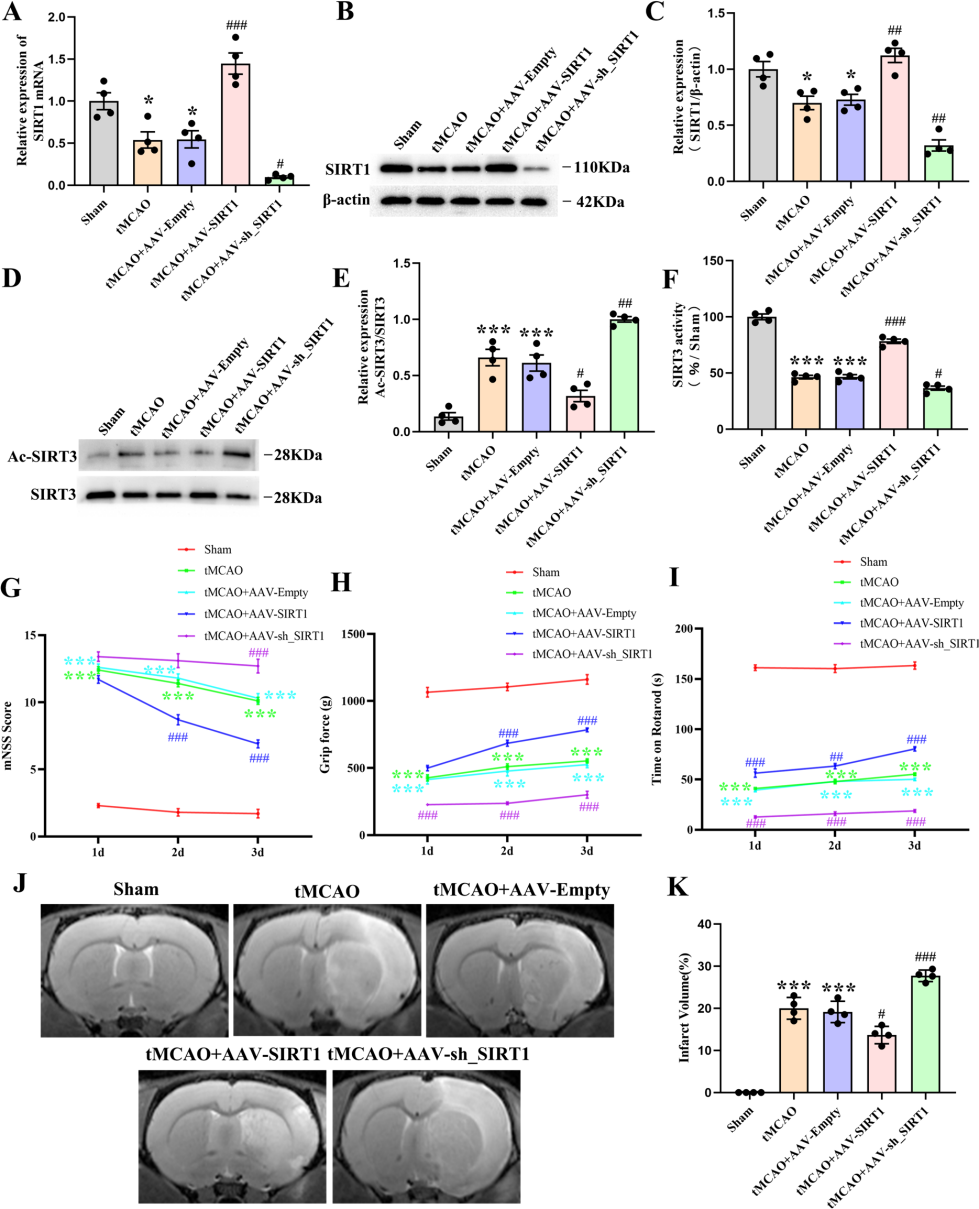
The effect of SIRT1 on the neurological function, infarct volume, and the acetylation and activity of SIRT3 in rats after tMCAO. **A** qPCR analysis of the SIRT1 level; *n* = 4. **B** Immunoblot analysis and (**C**) Quantification of SIRT1 level; *n* = 4. **D** Immunoblot analysis and (**E**) Quantification of the ratio of Ac-SIRT3/SIRT3; *n* = 4. **F** Quantification of SIRT3 activity; *n* = 4. **G** mNSS score, **H** grip strength test, **I** rotarod test; *n* = 10. **J** T2-weighted MRI representative images and (**K**) Quantification of the infarct volume; *n* = 10

To illustrate the effect of SIRT1 on the acetylation and activity of SIRT3, AAV-SIRT1 and AAV-sh_SIRT1 were locally injected into the cortex and striatum of rats. The analyses revealed that the protein and mRNA expression of SIRT1 declined in the tMCAO group and the AAV-Empty-treated group when in comparison with that of the untreated rats, but increased in the rats receiving SIRT1 overexpression and decreased in the rats receiving SIRT1 interference when compared with that of rats treated with AAV-Empty (Fig. 2A-C).

We subsequently assessed the involvement of SIRT1 in the acetylation and activity of SIRT3 in rats. The results indicated that compared with rats treated with AAV-Empty, the acetylation of SIRT3 was notably reduced by SIRT1 overexpression and increased by SIRT1 interference (Fig. 2D, E). Additionally, the activity of SIRT3 was significantly enhanced by SIRT1 overexpression but weakened by SIRT1 interference (Fig. 2F). Altogether, these results demonstrate that SIRT1 can decrease the acetylation of SIRT3 and enhance the activity of SIRT3 after CI/R.

Next, the impact of SIRT1 on neurological and motor function was evaluated in rats at 1d, 2d, and 3d after tMCAO by mNSS scoring, the grip strength test, and rotarod test, respectively. The mNSS scores indicated that compared with the AAV-Empty-treated rats, SIRT1 overexpression improved neurologic functional recovery after tMCAO and SIRT1 interference aggravated the neurological dysfunction after tMCAO (Fig. 2G). Likewise, the grip strength of the rats treated with SIRT1 overexpression was significantly improved when compared with those treated with AAV-Empty. Conversely, the grip strength of rats receiving SIRT1 interference was decreased when compared with those treated with AAV-Empty (Fig. 2H). Consistently, the rotarod test demonstrated that the time of staying on the rod was prolonged by SIRT1 overexpression but shortened by SIRT1 interference when compared with that of the AAV-Empty-treated rats (Fig. 2I). Furthermore, MRI examination revealed an obvious brain infarction in rats treated with tMCAO and those treated with AAV-Empty at 3d after tMCAO, which was markedly diminished by SIRT1 overexpression but expanded by SIRT1 interference (Fig. 2J, K). Collectively, these findings evidence that SIRT1 can offer substantial neuroprotection against C/R-induced neural damage.

### SIRT1 mitigates OGD/R-induced neuronal apoptosis in vitro and inhibits CI/R-induced apoptosis in vivo

**Fig. 3:**
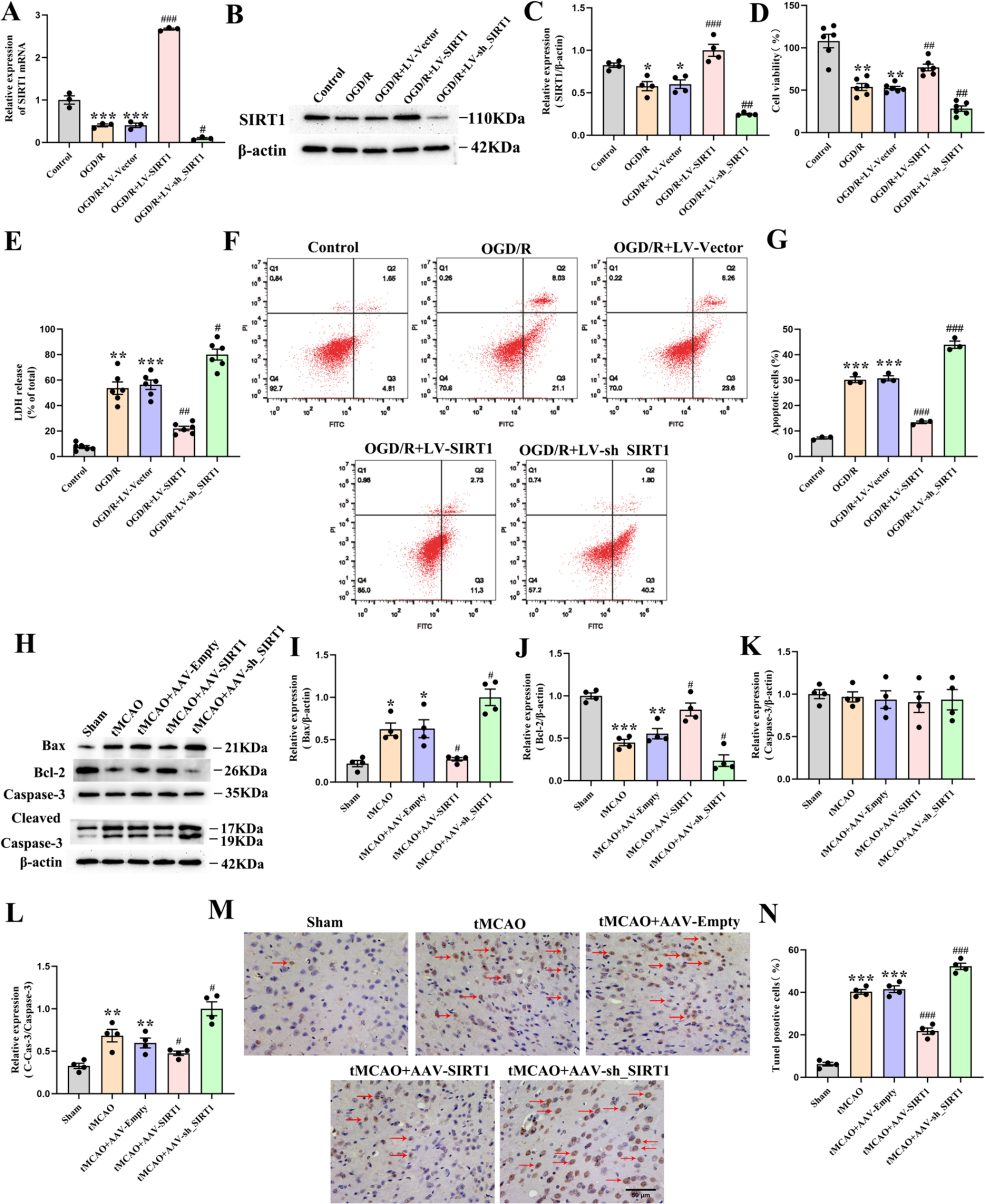
The amelioration of OGD/R induced-neuronal apoptosis in vitro and CI/R-induced apoptosis in vivo by SIRT1. **A** qPCR analysis of the SIRT1 level in the neurons; *n* = 3. **B** Immunoblot analysis and (**C**) quantification of SIRT1 level in the neurons; *n* = 3. **D** Cell viability; *n* = 6. **E** Cell cytotoxicity; *n* = 6. **F**, **G** The apoptotic percentage in neurons by flow cytometry; *n* = 3. **H** Immunoblot analysis and (**I**) quantification of Bax, **J** Bcl-2, **K** Cas-3 and (**L**) C-Cas-3 levels, *n* = 4. **M** Tunel staining representative images and (**N**) the proportion of Tunel-positive cells; *n* = 3, scale bar:50 um

To further validate the impact of SIRT1 on cell apoptosis after OGD/R injury, LV-SIRT1 and LV-sh_SIRT1 were respectively transfected into cultured neurons. The analysis showed that the protein and mRNA levels of SIRT1 were upregulated by LV-SIRT1 transfection but downregulated by LV-sh_SIRT1 transfection (Fig. 3A-C). In addition, compared with LV-Vector-transfected neurons, LV-SIRT1 transfection promoted cell viability and decreased LDH release, which was reversed by LV-sh_SIRT1 transfection (Fig. 3D, E). Compared with LV-Vector-transfected neurons, the apoptotic percentage in the cultured primary neurons was significantly decreased by LV-SIRT1 transfection but increased by LV-sh_SIRT1 transfection (Fig. 3F, G). These results collectively indicate that SIRT1 alleviates neuronal cell death caused by OGD/R.

The impact of SIRT1 on neuronal apoptosis was further investigated in rats following tMCAO. The results showed that the tMCAO-treated rats and those treated with AAV-Empty reported a marked downregulation in the expression of Bcl-2, and a significant upregulation in the expressions of Bax and Cleaved-Caspase-3 (C-Cas-3) when in comparison with the untreated rats. Compared with the AAV-Empty-treated rats, SIRT1 interference downregulated the expression of Bcl-2 but upregulated the expressions of C-Cas-3 and Bax, which was reversed by SIRT1 overexpression. Nevertheless, no significant alteration in Caspase-3 (Cas-3) levels was evident across the groups (Fig. 3H-L). Consistent with the above results, compared with the AAV-Empty-treated rats, the proportion of Tunel-positive cells was decreased by SIRT1 overexpression but increased by SIRT1 interference (Fig. 3M, N). Altogether, these results demonstrate that SIRT1 protects the brain against CI/R injury-induced apoptosis.

### SIRT1 alleviates mitochondrial oxidative stress and improves mitochondrial biogenesis in vivo and in vitro

**Fig. 4:**
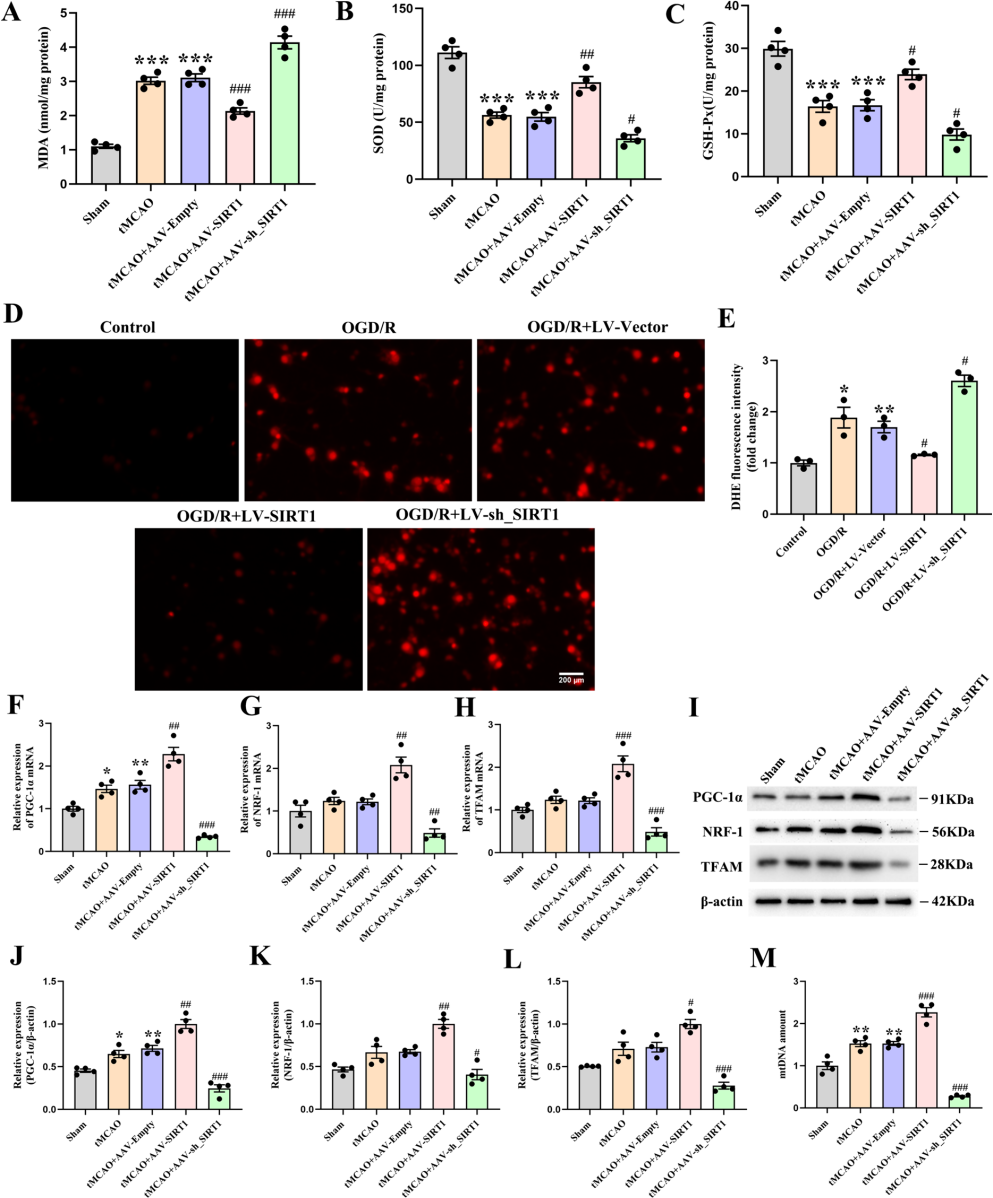
The alleviated mitochondrial oxidative stress and improved mitochondrial biogenesis in vitro and in vivo after CI/R injury by SIRT1. **A** The level of MDA, **B** the activity of SOD and (**C**) GSH-Px; n = 3. **D** DHE representative fluorescence images and (**E**) quantification of fluorescence intensity in neurons; *n* = 3. **F** qPCR analysis of the PGC-1α, **G** NRF-1, and (**H**) TFAM levels; *n* = 4. **I** Immunoblot analysis and (**J**) quantification of the PGC-1α, **K** NRF-1 and (**L**) TFAM levels; *n* = 4. **M** qPCR analysis of the relative mtDNA amount; n = 4

The oxidative stress-related biochemical markers were then examined after CI/R injury. The analysis demonstrated that compared with the untreated rats, the tMCAO-treated rats and those treated with AAV-Empty showed an increase in the MDA but a decrease in the activity of GSH-Px and SOD; compared with the AAV-Empty-treated rats, SIRT1 overexpression decreased the MDA level and increased the activity of GSH-Px and SOD, which was reversed by SIRT1 interference (Fig. 4A-C). As accumulated ROS in mitochondria is the dominant source of intracellular oxidative stress, we next detected the generation of ROS in the cultured primary cortical neurons. In relation to the untreated neurons, OGD/R induced a ROS overproduction, featuring an increase in DHE fluorescence intensity. Compared with the LV-Vector-transfected neurons, the DHE fluorescence intensity was reduced by LV-SIRT1 transfection but enhanced by LV-sh_SIRT1 transfection (Fig. 4D, E). Altogether, these results evidence that after CI/R, SIRT1 alleviates the in vitro and in vivo damage from mitochondrial oxidative stress.

We further investigated the impact of SIRT1 on mitochondrial biogenesis following CI/R. The analysis indicated that compared with the untreated rats, the tMCAO-treated rats and those treated with AAV-Empty reported a mild increase in the protein and mRNA expression of PGC-1α, with no alteration found in either NRF-1 or TFAM. In comparison with the AAV-Empty-treated rats, the protein and mRNA expressions of PGC-1α, NRF-1 and TFAM were markedly upregulated by SIRT1 overexpression but downregulated by SIRT1 interference (Fig. 4F-L). Next, the mtDNA content was further detected. Compared with the untreated rats, the tMCAO-treated rats and those treated with AAV-Empty reported an increase in the mtDNA content. Compared with the AAV-Empty-treated rats, the mtDNA content was increased by SIRT1 overexpression but decreased by SIRT1 interference (Fig. 4M). Altogether, these results evidence that SIRT1 can significantly improve mitochondrial biogenesis after CI/R.

### SIRT1 retains the mitochondrial integrity and alleviates the damage to the mitochondrial morphology after OGD/R or CI/R

**Fig. 5:**
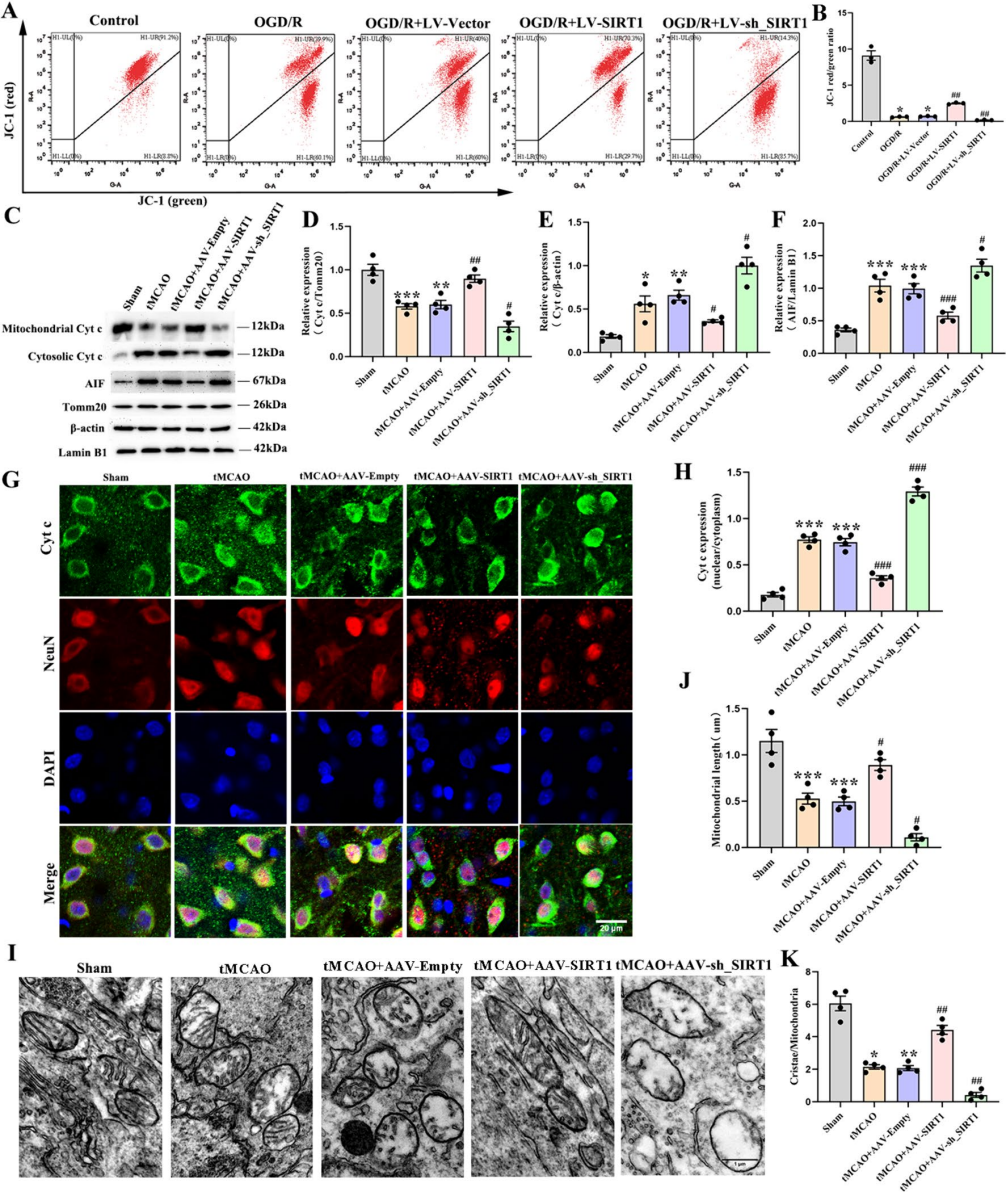
The retained mitochondrial integrity and alleviated mitochondrial morphology damage after OGD/R or CI/R by SIRT1. **A** JC-1 expression analyzed by flow cytometry and (**B**) quantification of JC-1 expression; *n* = 3. **C** Immunoblot analysis and (**D**) quantification of the mitochondrial Cyt c level, **E** the cytosol Cyt c level, and (**F**) the nuclear AIF level; *n* = 4. **G** Representative fluorescence images of Cyt c and NeuN; Scale bar: 20 μm. **H** Quantification of Cyt c expression; *n* = 3. **I** Representative TEM pictures. Scale bar: 1 μm. **J** Mitochondrial length; 50–60 mitochondria per experiment. **K** Mitochondrial cristae number; 50–60 mitochondria per experiment

As the integrity of mitochondria is significantly disrupted as an initial response to ischemic damage, which is a crucial change, the role of SIRT1 in protecting the structural stability of mitochondrial membrane was subsequently explored. The analysis indicated that OGD/R exaggerated the depolarization of the mitochondrial membrane potential, featuring in neurons a notable increase in JC-1 monomer formation (green fluorescence) and a marked decrease in JC-1 aggregates (red fluorescence). However, in comparison with the LV-Vector-transfected neurons, the depolarization of the mitochondrial membrane potential was significantly reduced by SIRT1 overexpression and worsened by SIRT1 interference (Fig. 5A-B). In addition, compared with the untreated rats, the tMCAO-treated rats and those treated with AAV-Empty reported an upregulated protein level of nuclear AIF and cytosolic Cyt c, but a decrease in that of the mitochondrial Cyt c. Compared with the AAV-Empty-treated rats, SIRT1 overexpression notably downregulated the protein levels of nuclear AIF and cytosolic Cyt c, and upregulated that of the mitochondrial Cyt c, which was reversed by SIRT1 interference (Fig. 5C-F). Moreover, the immunofluorescence assay revealed that Cyt c was co-localized with NeuN and that Cyt c was dramatically diffused into the nucleus after CI/R, which was alleviated by SIRT1 overexpression but aggravated by SIRT1 interference (Fig. 5G, H). Altogether, the above data demonstrate that SIRT1 can beneficially preserve the integrity of the mitochondria.

Then, the ultrastructure of mitochondria was assessed by TEM at 3d after tMCAO. Compared with the untreated rats, the tMCAO-treated rats and those treated with AAV-Empty displayed a reduction in the mitochondrial length and the number of mitochondrial cristae. However, compared with the AAV-Empty-treated rats, SIRT1 overexpression elongated the mitochondrial length and increased the number of mitochondrial cristae, which was reversed by SIRT1 interference (Fig. 5I-K). Collectively, these results indicate that SIRT1 alleviates the damage to the mitochondrial morphology after CI/R.

### SIRT1 promotes mitochondrial respiratory function after OGD/R or CI/R

**Fig. 6:**
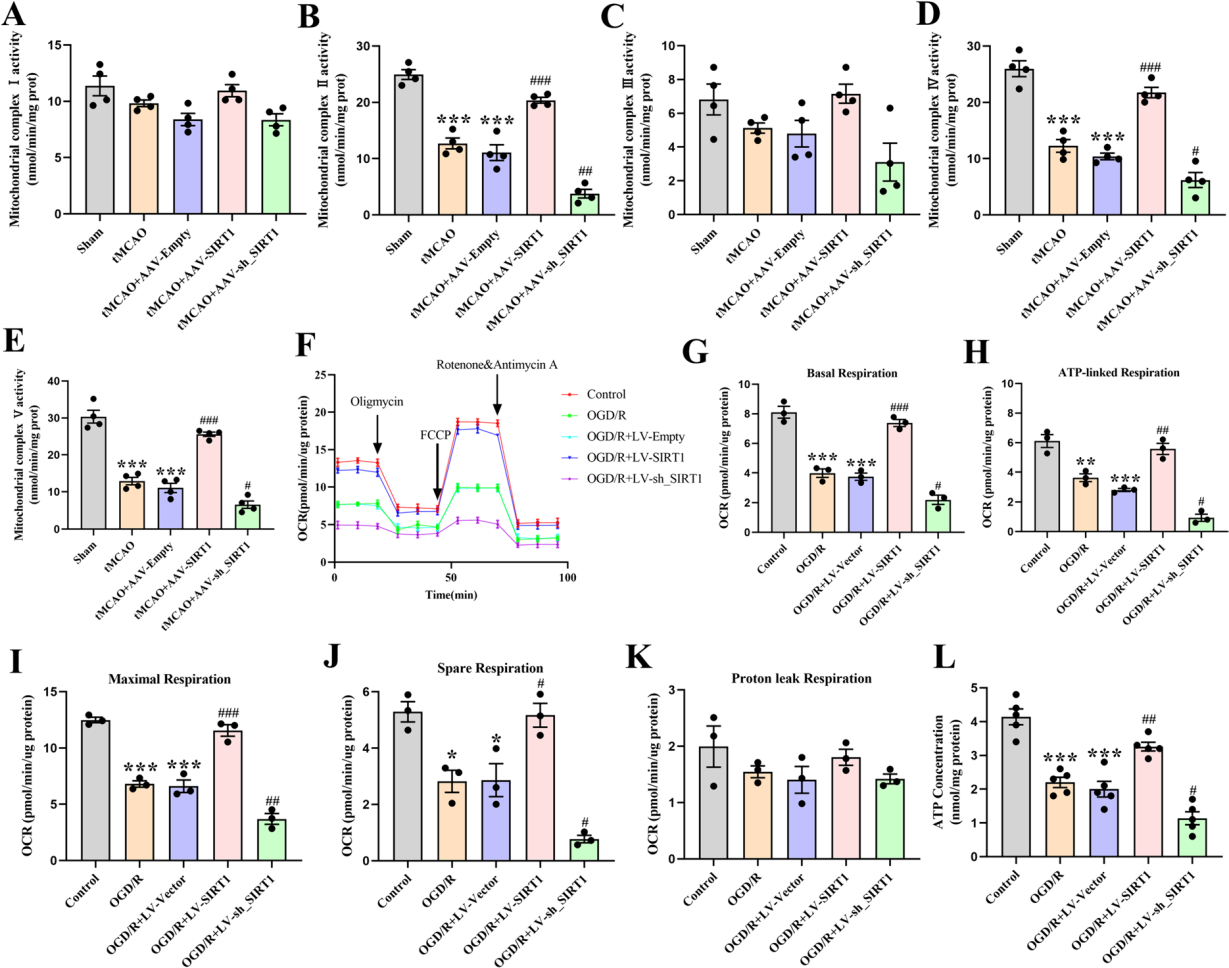
The alleviated mitochondrial respiratory dysfunction after OGD/R by SIRT1. **A**-**E** Quantification of complexes I-V; *n* = 3. **F** Representative OCR profile. **G** Basal respiration, **H** ATP-linked respiration, **I** maximal respiration, **J** spare respiration and (**K**) proton leak respiration; *n* = 3. **L** Quantification of ATP levels in neurons; *n* = 3

The activities of five mitochondrial complexes were then detected in the cortex. The analysis showed a decrease in the activities of complexes II, IV, and V in the tMCAO-treated rats and those treated with AAV-Empty. Compared with the AAV-Empty-treated rats, the activities of complexes II, IV, and V were increased by SIRT1 overexpression but decreased by SIRT1 interference. However, no distinguishable differences were observed in complexes I and III among these groups (Fig. 6A-E). We further assessed the effect of SIRT1 on OCR and ATP production in the neurons after OGD/R. Compared with the untreated neurons, the OGD/R-exposed and LV-Vector-transfected neurons displayed a decrease in the basal respiration, maximal respiration, ATP-linked respiration, and spare respiration. However, when in comparison with the neurons transfected with LV-Vector, an increase and a decrease in these specific respirations were respectively observed in the LV-SIRT1-transfected and LV-sh_SIRT1-transfected neurons (Fig. 6F-J). Nevertheless, no obvious change in proton leak respiration was evident among the groups (Fig. 6K). Moreover, OGD/R significantly decreased ATP contents in neurons. Compared with the LV-Vector-transfected neurons, the ATP content was significantly increased by LV-SIRT1 transfection but decreased by LV-sh_SIRT1 transfection (Fig. 6L). The above findings demonstrate that SIRT1 improves mitochondrial respiratory function after OGD/R.

## Discussion

In the present study, we assessed the involvement of SIRT1 in mitochondrial functional recovery and structural repair in rats undergoing CI/R exposure and explored the related molecular mechanisms. We found that SIRT1 promoted neurological functional recovery, mitigated CI/R or OGD/R-induced neuronal apoptosis, and ameliorated mitochondrial dysfunction by enhancing SIRT3 activity. These findings suggest that SIRT1 may potentially act as a valuable alternative for treating ischemic stroke and highlight the importance of exploring novel therapeutic strategies ischemic stroke.

SIRT1, a nuclear-cytoplasmic shuttling protein, is located in the nucleus and mitochondria (Aquilano et al. 2010). It exerts important cytoprotective effects by deacetylating histones and gene regulatory proteins (Jeninga et al. 2010; Prola et al. 2017; J Zhang et al. 2020). SIRT3 is a deacetylase in mitochondria that regulates mitochondrial function (Salvatori et al. 2017). Consistently, our study evidenced the presence of SIRT3 in mitochondria and SIRT1 in the cerebral nucleus and mitochondria. We further investigated whether SIRT1 interacted with SIRT3 in the cerebral mitochondria. The MD simulation reported a strong and stable binding between SIRT1 and SIRT3. Notably, the free energy analysis showed that the strength of the bond was influenced by the presence of both hydrogen bonding and hydrophobic interactions. This binding stability was verified in immunofluorescence experiments.

Available literature demonstrates that the deacetylation of SIRT3 can influence the activity of SIRT3 (Kwon et al. 2017; Yi et al. 2019). The latter can be significantly enhanced by SIRT1 overexpression in a hypoxia culture of human pulmonary arteriole smooth muscle cells (P Li et al. 2017a, b). SIRT1 overexpression has also been documented to significantly decrease the hyperacetylation of SIRT3 and enhance the activity of SIRT3 in obesity and aging-related diseases (Kwon et al. 2017). Consistently, in the current study, SIRT1 overexpression decreased the acetylation of SIRT3, enhancing the activity of SIRT3, which was reversed by SIRT1 interference, indicating that SIRT1 can enhance the activity of SIRT3 by reducing the acetylation of SIRT3. Collectively, these findings evidence that the function of mitochondria is crucial in alleviating CI/R-induced neuronal damage and that the “SIRT1/SIRT3 acetylation and SIRT3 activity” may be exploited for the treatment of ischemic stroke.

CI/R may incur severe damage to the neurological functions and brain tissues (Xu et al. 2019). After CI/R injury, the deterioration in asymmetry in forelimb use, muscle weakness, and motor coordination may occur. Therefore, we examined how SIRT1 affects the neurological function of rats experiencing CI/R injury by assessing their mNSS scores, grip strength, and rotarod performance. The results showed that SIRT1 overexpression alleviated neurological deficits, improved the muscle strength and motor coordination, and diminished the cerebral infarction, while SIRT1 interference markedly aggravated neurological deficits, weakened the muscle strength, decreased motor coordination, and increased the volume of cerebral infarction. Then, on the basis of SIRT1 overexpression, an SIRT3 activity inhibitor (3-TYP) was adopted to verify whether SIRT1 exerts its neuroprotection via the enhancement of SIRT3 activity, which showed that the treatment with 3-TYP partially offset the beneficial effect of SIRT1 overexpression (Result 1, Figure. S1.). These findings demonstrate that SIRT1 can improve CI/R-induced neurobehavioral dysfunction by enhancing the activity of SIRT3 and lend further support to the notion that “SIRT1/ SIRT3 activity” may play a neuroprotective role in treating ischemic stroke.

After CI/R injury, apoptosis, characterized by DNA fragmentation, significantly contributes to neuronal death (Radak et al. 2017). A recent study evidences that SIRT1 overexpression inhibits NMDA-induced excitotoxicity in cultured cortical neurons by decreasing the LDH leakage rate and increasing the number and viability of living cells (X Yang et al. 2017). Consistently, our findings revealed that SIRT1 overexpression ameliorated neuronal apoptosis by promoting neuronal survival and reducing neuronal toxicity. During cell apoptosis, Bcl-2 protein family and Cas-3 are key molecules. The former consists of anti-apoptotic Bcl-2 and pro-apoptotic Bax, two key antagonistic proteins (Peña-Blanco and García-Sáez 2018). The latter usually exists as an inactive zymogen, namely, Pro-Caspase-3, which can be activated into C-Cas-3 to promote cell apoptosis (Broughton et al. 2009; Esteban-Fernández de Ávila et al. 2017). Available studies have documented SIRT1 as a promising molecular target in regulating apoptosis (Gao et al. 2022; Ma et al. 2021) and indicated that SIRT1 can protect hypoxic cardiomyocytes from apoptosis by downregulating C-Cas-3 and upregulating Bcl-2 (Luo et al. 2019). Accordingly, to validate the effect of SIRT1 on anti-apoptosis, the protein levels of Bax, Cas-3, C-Cas-3, Bcl-2, and the Tunel-positive cells were detected in tMCAO-exposed rats. Consistently, we found that SIRT1 overexpression increased the Bcl-2 expression and decreased the levels of active Caspase-3, Bax, and the Tunel-positive cells after tMCAO, which was respectively reversed by SIRT1 interference. The 3-TYP treatment partially reversed the anti-apoptosis effect of SIRT1 overexpression (Result 2, Figure. S2.). These findings suggest that SIRT1 can prevent CI/R-induced neuronal apoptosis by enhancing SIRT3 activity. The proposed “SIRT1/SIRT3 activity” may highlight a novel therapeutic perspective for the clinical management of ischemic stroke-related conditions.

Oxidative stress can exacerbate I/R-induced tissue injury, which results from an imbalance between oxidants and antioxidants in the body, leading to an excessive production of reactive oxygen species (ROS) during I/R (Sies 2015). The excess ROS production can induce severe damage to cellular protein, lipid, and DNA (Orellana-Urzúa et al. 2020). As a byproduct of lipid peroxidation, MDA serves as an index for oxidative stress (Cojocaru et al. 2013). Antioxidant enzymes such as SOD, GSH, play a pivotal role in the neuronal resistance to ROS-induced cell death (Z Yang et al. 2016). Available literature has shown that SIRT1 downregulation can increase ROS level in acute ischemia (Yan et al. 2020) and that SIRT1 upregulation can evidently decrease MDA level, increase the levels of GSH-Px and SOD in doxorubicin-induced liver injury (Song et al. 2019). Likewise, we found that SIRT1 overexpression downregulated the levels of MDA and ROS, enhanced the activities of GSH-Px and SOD in cerebral ischemia, which were respectively reversed by SIRT1 interference. The anti-oxidative stress effect of SIRT1 overexpression was partially abolished by the 3-TYP treatment (Result 3, Figure. S3 A-E.). Collectively, these findings evidence that SIRT1 may provide neuroprotection against CI/R-induced oxidative stress by enhancing SIRT3 activity, signifying that a proper manipulation of “SIRT1/ SIRT3 activity” may alleviate the excessive oxidative stress in ischemic stroke.

Mitochondrial biogenesis, the production of new mitochondria from existing mitochondria (Popov 2020), is modulated by the signaling pathway of PGC-1α, NRF-1, and TFAM, the activation of which can lead to the synthesis of mtDNA and mitochondrial proteins (Gong et al. 2017). A recent study reports an elevation in the mRNA expression of PGC-1α and TFAM, but no significant change in that of NRF-1 in rats after ischemic stroke (Q Zhang et al. 2012). However, in the current study, the mRNA expression of NRF-1 remained unchanged following CI/R injury. The difference may be contributed to the different reperfusion time points in rats. A previous study has found that SIRT1 can enhance mitochondrial biogenesis in counteracting the neurotoxicity of developmental fluoride by initiating the PGC-1α/NRF-1/TFAM signaling pathway (Zhao et al. 2020). Another study has reported that the upregulation of SIRT1 can increase the mitochondrial DNAs in neonatal mouse cardiomyocytes (A J Liu et al. 2017). Consistently, the current study found that SIRT1 overexpression upregulated the expressions of PGC-1α, NRF-1, and TFAM, stimulating the synthesis of mtDNA, which was reversed by SIRT1 interference. The treatment with 3-TYP partially offset the beneficial effect of SIRT1 overexpression (Result 3, Figure. S3 F-I.). These results indicate that SIRT1 improves mitochondrial biogenesis by enhancing SIRT3 activity. These findings shed some new lights on the repairing mechanisms involved in ischemic stroke-induced cerebral damage and signify potential strategies in mitigating the negative impact of ischemia on mitochondrial function.

Mitochondrial permeability transition pore (mPTP) is a high-conductance channel in the mitochondrial integrity (R Li et al. 2020a, b, c, d), which can be opened by hypoxia or ischemia (V et al. 2021). The opening of mPTP can trigger the movement of Cyt c from the mitochondria into the cytoplasm, inducing the cellular apoptosis (Cowan et al. 2019). The cytoplasm influx of Cyt c further increases mitochondrial pro-apoptotic factor AIF, which can transfer from cytosol to nucleus, resulting in cell death (W H Li et al. 2020a, b, c, d). In our study, mPTP opening appeared in mitochondria after CI/R, which induced the decrease of membrane potential and the discharge of AIF and Cyt c. Nevertheless, these effects were markedly offset after the administration of AAV-SIRT1 and aggravated after the administration of AAV-sh_SIRT1. The treatment with 3-TYP partially reversed the beneficial effect of SIRT1 overexpression (Result 4, Figure. S4 A-F.). Together, these data suggest that after CI/R injury, SIRT1 improves mitochondrial integrity by enhancing SIRT3 activity. Therefore, the “SIRT1 SIRT3 activity” may provide novel insights into the potential strategies in safeguarding mitochondrial integrity against ischemic damage.

The adaptation of mitochondria to various physiological conditions depends strongly on the regulation of their structure, especially in the cristae compartment (Quintana-Cabrera et al. 2018). After CI/R, the mitochondrial structure is obviously disrupted, resulting in severe neurological dysfunction. Our study demonstrated that SIRT1 overexpression increased the mitochondrial length and mitochondrial cristae number after cerebral ischemia, which were respectively reversed by SIRT1 interference. The 3-TYP treatment partially offset the beneficial effect of SIRT1 overexpression (Result 4, Figure. S4 G-I.). Collectively, these data reveal that SIRT1 alleviates the CI/R-induced damage to the mitochondrial ultrastructure by enhancing SIRT3 activity and may have therapeutic potential for reducing mitochondrial structural damage from ischemic stroke.

The mitochondrial oxidative phosphorylation system plays a central role in cellular metabolism. It consists of five enzymatic complexes and two mobile electron carriers that function in a mitochondrial respiratory chain. This electron transport chain is responsible for generating ATP (Vercellino and Sazanov 2022). A previous study suggests that the energy failure of CI/R injury is associated with the decreased activity of respiratory complexes I – IV (Liu et al. 2013). In the present study, CI/R decreased the activities of complexes II, IV, V and the level of ATP. The inconsistencies may be explained by the variability in the activities of mitochondrial complexes in different regions. We also found that SIRT1 overexpression increased the activities of complexes II, IV, V and the level of ATP, which was reversed by SIRT1 interference. These results suggest that after CI/R injury, the ATP level may be influenced by the enhanced activity of the mitochondrial respiratory chain complex enzymes. Additionally, existing studies document that the ultrastructure of mitochondria affects respiratory function (Cogliati et al. 2013) and that the respiratory capacity and cell viability are dictated by the changes in cristae number and shape (Hackenbrock 1968; Varanita et al. 2015), in which the shift of the mitochondrial cristae shape from an orthodox to a condensed state mirrors a transition from a low respiratory status to a high respiratory status (Jayashankar et al. 2016). In our study, OCR data showed that SIRT1 retained mitochondrial bioenergetics by reversing the depletion of ATP and the OGD/R-induced decrease in related bioenergetic parameters. We speculate that after CI/R injury, the ATP level may parallel the increased activity of the enzymes of mitochondrial respiratory chain complex. The 3-TYP treatment partially abolished the beneficial effect of SIRT1 overexpression (Result 5, Figure. S5.). Altogether, these findings suggest that SIRT1 improves mitochondrial respiratory function by enhancing SIRT3 activity, which may illuminate the mechanism underlying the promotion of energy metabolism in treating ischemic stroke.

While our study sheds some light on the impacts of SIRT1 expression on acute ischemic stroke, some limitations remain. To gain a more comprehensive understanding, further research is awaited to investigate the long-term neurological and mitochondrial function recovery in cultured primary neurons and in ischemic rats. Additionally, this study mainly observed the acetylation and activity of SIRT3 by up-regulating or down-regulating the expression of SIRT1 in ischemic rats. However, future investigation is desired to illuminate the specific mechanism by which SIRT3 acetylation affects SIRT3 activity. The present study evidences that SIRT1 improves neurological recovery and attenuates neuronal apoptosis and mitochondrial dysfunction after CI/R injury by enhancing SIRT3 activity. These findings provide a theoretical basis for the proposed therapeutic value of “SIRT1/ SIRT3 activity” in stroke prevention and treatment.

## Supplementary Information

Below is the link to the electronic supplementary material.

Supplementary file1 (DOCX 3.72 MB)

Supplementary Figure 7 (PNG 561 kb)

(TIF 1.09 MB)

## Data Availability

The datasets used and/or analysed during the current study are available from the corresponding author on reasonable request.

## Abbreviations

*CI/R*: Cerebral ischemia–reperfusion
*SIRT3*: Sirtuin-3
*SIRT1*: Sirtuin-1
*SD*: Sprague-Dawley
*AAV-SIRT1*: Adeno-associated viral Sirtuin-1
*tMCAO*: Transient middle cerebral artery occlusion
*LV-SIRT1*: Lentiviral SIRT1
*OGD/R*: Oxygen-glucose deprivation/reoxygenation
*3-TYP*: 3-(1H-1,2,3-triazol-4-yl) pyridine
*TEM*: Transmission electron microscopy
*MRI*: Magnetic resonance imaging
*MD*: Molecular dynamics
*ROS*: Reactive oxygen species
*mPTP*: Mitochondrial permeability transition pore
*ΔΨm*: Mitochondrial membrane potential
*Cyt c*: Cytochrome c
*mtDNA*: Mitochondrial DNA
*A**TP*: Adenosine triphosphate
*I/R*: Ischemia-reperfusion
*PASMCs*: Pulmonary arteriolar smooth muscle cells
*H/R*: Hypoxia/reoxygenation
*CCA*: Common carotid artery
*ICA*: Internal carotid artery
*ECA*: External carotid artery
*mNSS*: Modified neurological severity score
*ST*: Slices thickness
*FOV*: Field of view
*TR*: Time of repetition
*TE*: Time of echo
*qRT-PCR*: Real-time quantitative PCR
*IP*: Immunoprecipitation
*CCK-8*: Cell counting kit-8
*LDH*: Lactate Dehydrogenase
*OCR*: Oxygen consumption rate
*Tunel*: Terminal dUTP nick-end labelling
*MDA*: Malondialdehyde
*SOD*: Superoxide dismutase
*GSH-Px*: Glutathione peroxidase
*BSA*: Bovine serum albumin
*RMSD*: Root-mean-square deviation
*Rg*: Radius of gyration
*RMSF*: Root-mean-square fluctuations
*SASA*: Solvent accessible surface area
*HBnum*: Hydrogen bond number
*SEM*: Standard error of the mean
*C-Cas-3*: Cleaved-Caspase-3
*Cas-3*: Caspase-3
