# Supplementary material for: SIRT1 restores mitochondrial structure and function in rats by activating SIRT3 after cerebral ischemia/reperfusion injury
Source: Cell Biol Toxicol. 2024 May 20;40(1):31. doi: 10.1007/s10565-024-09869-2 (PMC11106166; doi:10.1007/s10565-024-09869-2)
Supplement: Supplementary file 1 — Supplementary file1 (DOCX 3.72 MB) [file 10565_2024_9869_MOESM1_ESM.docx]

**Materials and Methods**

**1. Assessment of the activity of SIRT3 and SIRT1**

As per the instructions from the manufacturer, the activities of SIRT3 and SIRT1 were respectively determined using the SIRT3 Fluorometric Drug Discovery Kit and SIRT1 Fluorometric Drug Discovery assay kit (BML-AK557-0001, BML-AK555-0001; both from Enzo Life Sciences, USA). The activities of SIRT3 and SIRT1 were spectrophotometrically determined with a SpectraMax i3x (SpectraMax®i3x, Molecular Devices, USA).

**2. TdT-mediated dUTP nick end labelling (Tunel) staining**

The apoptosis of cortical tissue in the peri-ischemic area was measured with an In Situ Cell Death Detection Kit (Cat# 11684817910, Roche Applied Science, Penzberg, Germany) as previously described with minor modification (Xu et al., 2020). Briefly, serial sections were deparaffinized, rehydrated, and digested before receiving 50 ul of the Tunel reaction mixture. Next, the samples were dried, and received 50 ul of the transforming agent, POD. Finally, the Tunel reaction was performed with a fresh 3, 3-diaminobenzidine (DAB) solution and the Tunel-positive cells were identified with hematoxylin by counter-staining. Tissues were visualized under a fluorescence microscope with a 40 × objective (Olympus, Tokyo, Japan).

**3. Transmission electron microscopy (TEM)**

As previously described, the changes in the mitochondrial ultrastructure were observed by TEM at 3 d after tMCAO (Zhang et al., 2021). The cortical tissue in the peri-ischemic area was quickly removed and sliced into particles (1 mm3 in size). Then, the samples were stained with uranyl acetate and lead citrate, and visualized by TEM (EM208, Philips, Eindhoven, the Netherlands).

**4. Detection of mitochondrial membrane potential and cell apoptosis**

As per the instructions from the manufacturer, the alterations in mitochondrial membrane potential and cell apoptosis were assessed using a JC-1 Assay Kit (Cat# M34152, Thermo Fisher Scientific, MA, USA) and FITC-Annexin V apoptosis detection kit (Cat# 556547, BD Biosciences, CA, USA) respectively. After sample analysis through flow cytometry, the data was further analyzed using the FlowJo software.

**5. Determination of ROS**

As per the instructions from the manufacturer, the presence of reactive oxygen species (ROS) in viable cells was identified using a Dihydroethidium (DHE) dye (Product number S0033S, Beyotime, Shanghai, China). In brief, neurons seeded on glass coverslips underwent a wash with pre-heated PBS and were then exposed to 3 μM of DHE at 37℃ for 30 min. The neurons were observed under a confocal microscope (LSM 750, Zeiss, Gottingen, Germany).

**6. Immunoprecipitation (IP)**

As per the instructions from the manufacturer, an equal amount of anti-SIRT3 was added to the lysate for incubation on a shaker at 4℃ overnight. Then, the above extract EP tube received 20 ul of protein A/G magnetic beads. After a centrifugation of 1000g for 5 min, the precipitate was collected. Subsequently, the samples were resuspended in a loading buffer containing 1× SDS and boiled at 100℃ for 5 min. After centrifugation, the supernatant was obtained and utilized for the following immunoblotting experiments. The membranes were visualized with the chemiluminescence system (Bio-Rad, CA, USA).

**7. Detection of cytotoxicity and cell viability**

The cytotoxicity (Pérez-Mato et al., 2019) and cell viability (Che et al., 2019) were respectively assessed with an enhanced cell counting kit-8 (CCK-8) assay kit (Cat# CK04-11, Dojindo, Kumamoto, Japan) and Lactate Dehydrogenase (LDH) assay kit (Cat# MAK066-1 KT, Sigma-Aldrich, MO, USA). Finally, a multifunctional microplate reader (SpectraMax®i3x, Molecular Devices, USA) was used to measure the OD values of the samples (CCK-8: 490 nm; LDH: 450 nm).

**8. Quantification of mitochondrial DNA (mtDNA) content**

The total DNA was acquired with an EasyPure Genomic DNA Kit (Cat# CK04-11, Transgen, Beijing, China). As previously described (Al Rahim, Thatipamula, Pasinetti, & Hossain, 2021), qRT-PCR was performed to determine the amount of mtDNA in relation to nuclear genomic DNA. The primer sequences are detailed in Table S1. The copy number of mtDNA was quantified by the −2^ΔΔCt^ method.

**Results**

**1 SIRT1 improves the neurological function and attenuates the infarct volume in rats after CI/R by enhancing SIRT3 activity**

A selective SIRT3 inhibitor, 3-TYP, was utilized to investigate the involvement of SIRT3 activity in the impact of SIRT1 on mitochondrial structural repair and functional recovery. We first explored the impact of 3-TYP on SIRT1 expression and activity in vivo. The results revealed no noticeable change in the expression and activity of SIRT1 either in the tMCAO+3-TYP-treated rats when compared with the tMCAO-treated rats or in the tMCAO+AAV-SIRT1+3-TYP-treated rats when compared with the tMCAO+AAV-SIRT1-treated rats (Fig. S1A-C). Next, we examined the impact of 3-TYP on the acetylation and activity of SIRT3. The results indicated no obvious change in the acetylation level of SIRT3 either in the tMCAO+3-TYP-treated rats when compared with the tMCAO-treated rats or in rats treated with tMCAO+AAV-SIRT1+3-TYP when compared with the tMCAO+AAV-SIRT1-treated rats (Fig. S1D-E). However, the activity of SIRT3 was weakened both in the tMCAO+3-TYP-treated rats when compared with the tMCAO-treated rats and in rats treated with tMCAO+AAV-SIRT1+3-TYP when compared with the tMCAO+AAV-SIRT1-treated rats (Fig.S1F). These results demonstrate that although 3-TYP treatment exerts no influence on the activity and protein expression of SIRT1 and the acetylation level of SIRT3, it strongly inhibits the activity of SIRT3 after CI/R.

To investigate whether SIRT3 activity participates in the neuroprotective effects of SIRT1, mNSS, the grip strength test, and rotarod test were further performed. The results showed that compared with the tMCAO-treated rats, the tMCAO+3-TYP-treated rats reported a significant increase in mNSS scores after tMCAO. A similar mNSS increase was observed in rats treated with tMCAO+AAV-SIRT1+3-TYP at 3d when compared with the tMCAO+AAV-SIRT1-treated rats (Fig. S1G). Likewise, compared with the tMCAO-treated rats, the tMCAO+3-TYP-treated rats reported a reduced grip strength and a shortened stay on the rod after tMCAO. Similar changes were found in rats treated with tMCAO+AAV-SIRT1+3-TYP as compared with the tMCAO+AAV-SIRT1-treated rats (Fig. S1H-I). Furthermore, MRI showed that the brain infarct volume in the tMCAO+3-TYP-treated rats was significantly enlarged when compared with the tMCAO-treated rats. A similar change was evident in the rats treated with tMCAO+AAV-SIRT1+3-TYP when compared with the tMCAO+AAV-SIRT1-treated rats (Fig. S1J-K). These results demonstrate that SIRT1 can improve the neurological function and attenuate the infarct volume in rats after CI/R, which can be partially abolished by 3-TYP.

**
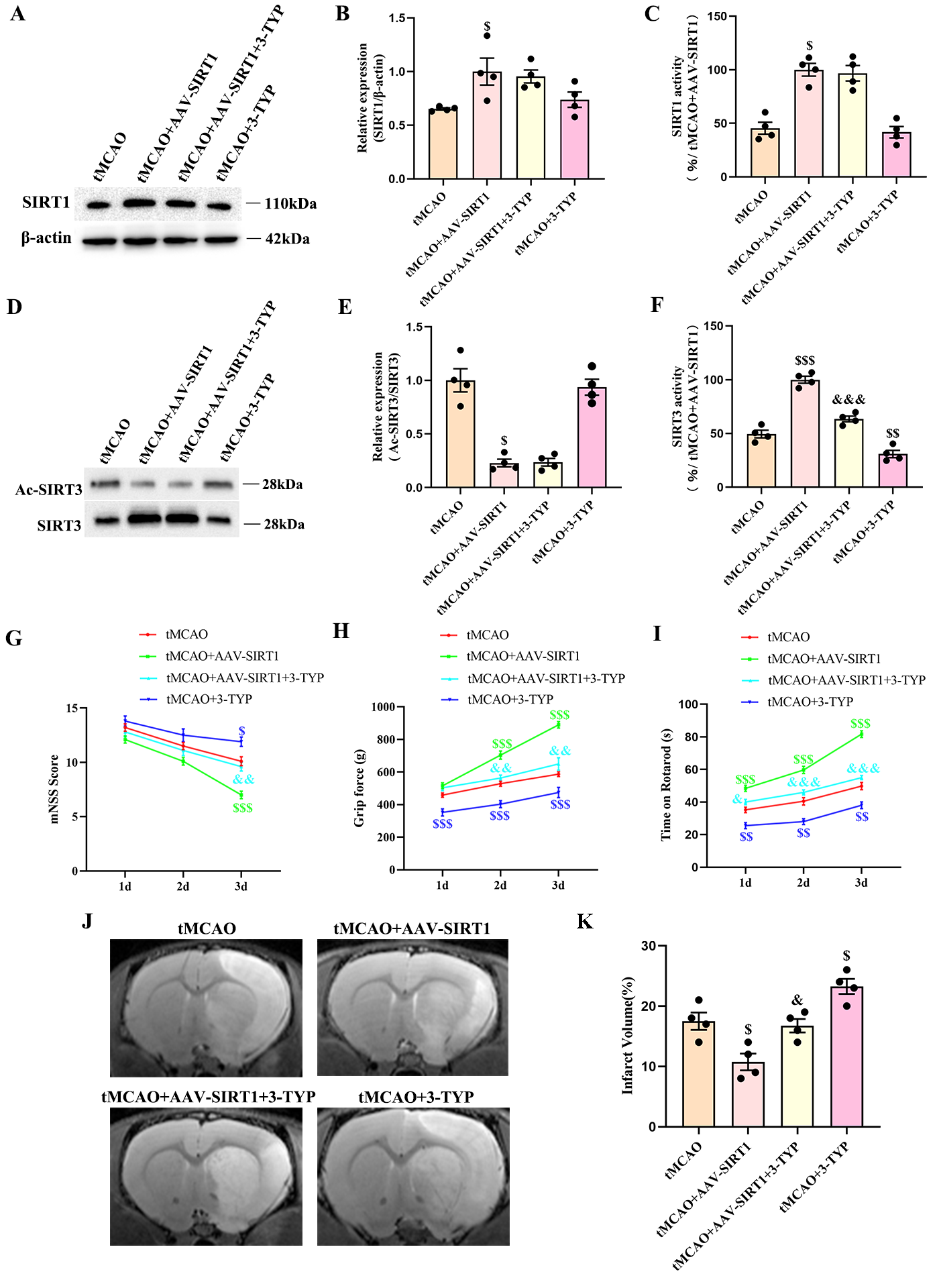
**

**Fig.S1. The improved neurological function and reduced infarction in rats after CI/R injury by SIRT1 via the enhancement of SIRT3 activity**. (A) Immunoblot analysis and (B) quantification of SIRT1 level; n = 4. (C) Quantification of SIRT1 activity; n = 4. (D) Immunoblot analysis and (E) quantification of the ratio of Ac-SIRT3/SIRT3; n = 4. (F) Quantification of SIRT3 activity; n = 4. (G) mNSS score, (H) grip strength test, and(I) rotarod test; n=10. (J) T2-weighted MRI representative images and (K) quantification of the infarct volume; n = 10.

**2 SIRT1 alleviates the apoptotic effects after CI/R or OGD/R by enhancing SIRT3 activity**

We then examined the involvement of SIRT3 in the SIRT1-mediated anti-apoptosis after CI/R. The results showed that in comparison with the tMCAO-treated rats, the tMCAO+3-TYP-treated rats reported an increase in the expressions of C-Cas-3 and Bax but a decline in the expression of Bcl-2. Similar changes were evident in the tMCAO+AAV-SIRT1+3-TYP-treated rats when compared with the tMCAO+AAV-SIRT1-treated rats (Fig. S2A-E). In addition, when in comparison with the tMCAO-treated rats, the tMCAO+3-TYP-treated rats reported an increased percentage of Tunel-positive cells. A similar increase was found in rats treated with tMCAO+AAV-SIRT1+3-TYP when compared with the tMCAO+AAV-SIRT1-treated rats (Fig. S2F-G). Consistently, the flow cytometry further showed that the OGD/R+3-TYP-treated neurons reported an aggravated apoptosis when in comparison with the OGD/R-exposed neurons. A similar trend was present in the neurons treated with OGD/R+LV-SIRT1+3-TYP when in comparison with the OGD/R+LV-SIRT1-transfected neurons (Fig. S2H-I). These results suggest that SIRT1 alleviates the apoptotic effects after CI/R or OGD/R, which can be partially abolished by 3-TYP.


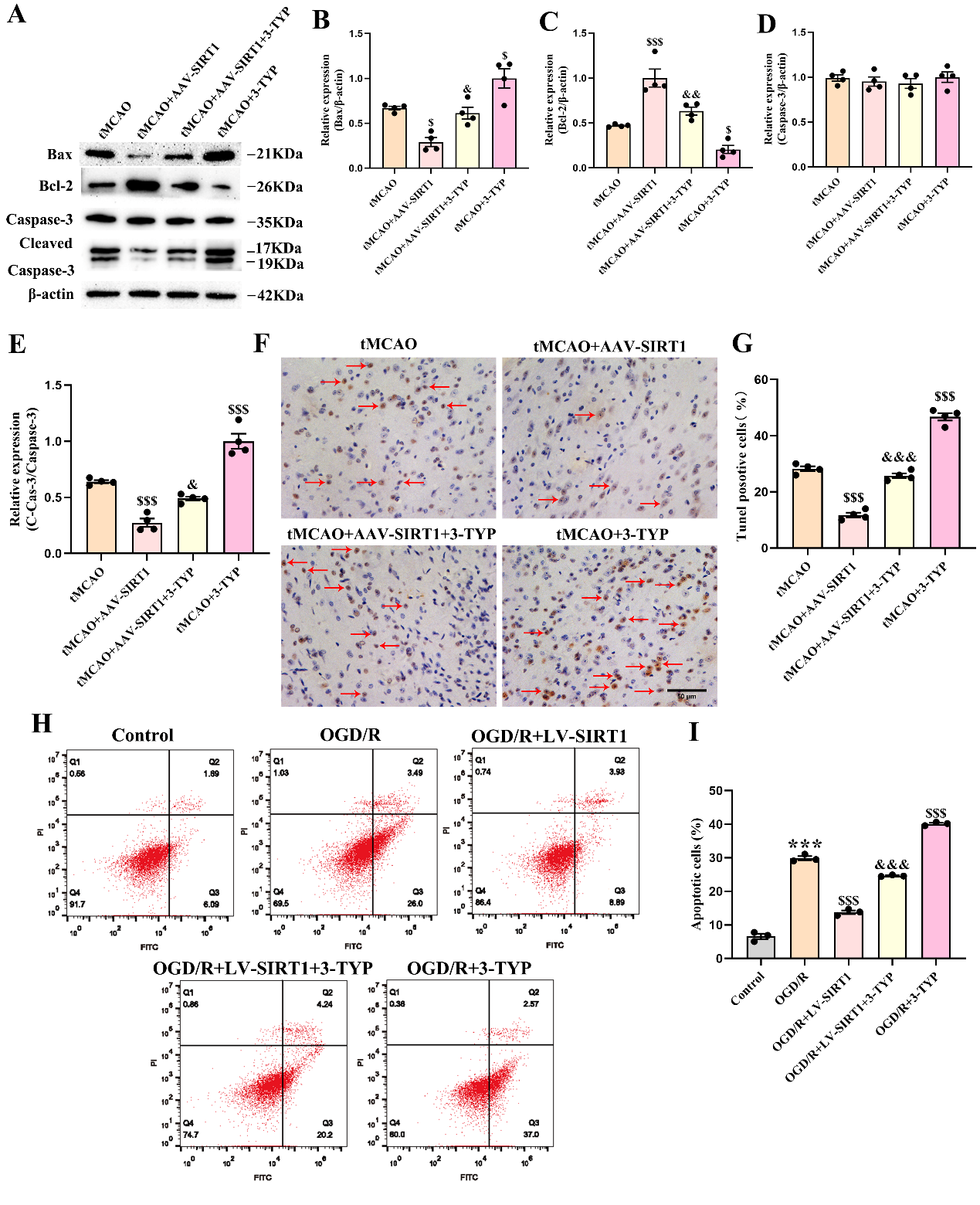


**Fig.S2. The alleviated apoptotic effects after CI/R or OGD/R by SIRT1 via the enhancement of SIRT3 activity.** (A) Immunoblot analysis and (B) quantification of Bax levels; n = 4. (C) Quantification of Bcl-2 levels; n=4. (D) Quantification of Cas-3, and (E) C-Cas-3 levels; n=4. (F) Representative images of Tunel staining and (G) the percentage of Tunel-positive cells; n = 4, Scale bar: 50 μm. (H, I) The apoptotic percentage in neurons by flow cytometry; n = 3.

**3 SIRT1 alleviates oxidative effects and improves mitochondrial biogenesis after CI/R or OGD/R by enhancing SIRT3 activity.**

We determined the roles of SIRT3 in the anti-oxidative effects of SIRT1 after CI/R. The results indicated that compared with the tMCAO-treated rats, the tMCAO+3-TYP-treated rats reported a decreased activity of SOD and GSH-Px but a increase in MDA. Similar changes were evident in the rats treated with tMCAO+AAV-SIRT1+3-TYP when compared with the rats treated with tMCAO+AAV-SIRT1 (Fig. S3A-C). The OGD/R+3-TYP-treated neurons reported an overproduction of ROS when in comparison with the OGD/R-exposed neurons. Similarly, when compared with the OGD/R+LV-SIRT1-treated neurons, the neurons treated with OGD/R+LV-SIRT1+3-TYP showed an overproduced level of ROS (Fig. S3D, E). These results indicate that SIRT1 alleviates oxidative effects after CI/R or OGD/R, which can be partially abolished by 3-TYP.

Further analyses were conducted to verify whether SIRT3 participates in the SIRT1-mediated protection of mitochondrial biogenesis after CI/R. The results indicated that the protein expressions of PGC-1α, NRF-1 and TFAM were downregulated in the tMCAO+3-TYP-treated rats when in comparison with the rats treated with tMCAO. Similar changes were reported in the rats treated with tMCAO+AAV-SIRT1+3-TYP when compared with the tMCAO+AAV-SIRT1-treated rats (Fig. S3F-I). These results indicate that SIRT1 improves mitochondrial biogenesis after CI/R, which can be partially abolished by 3-TYP.

**
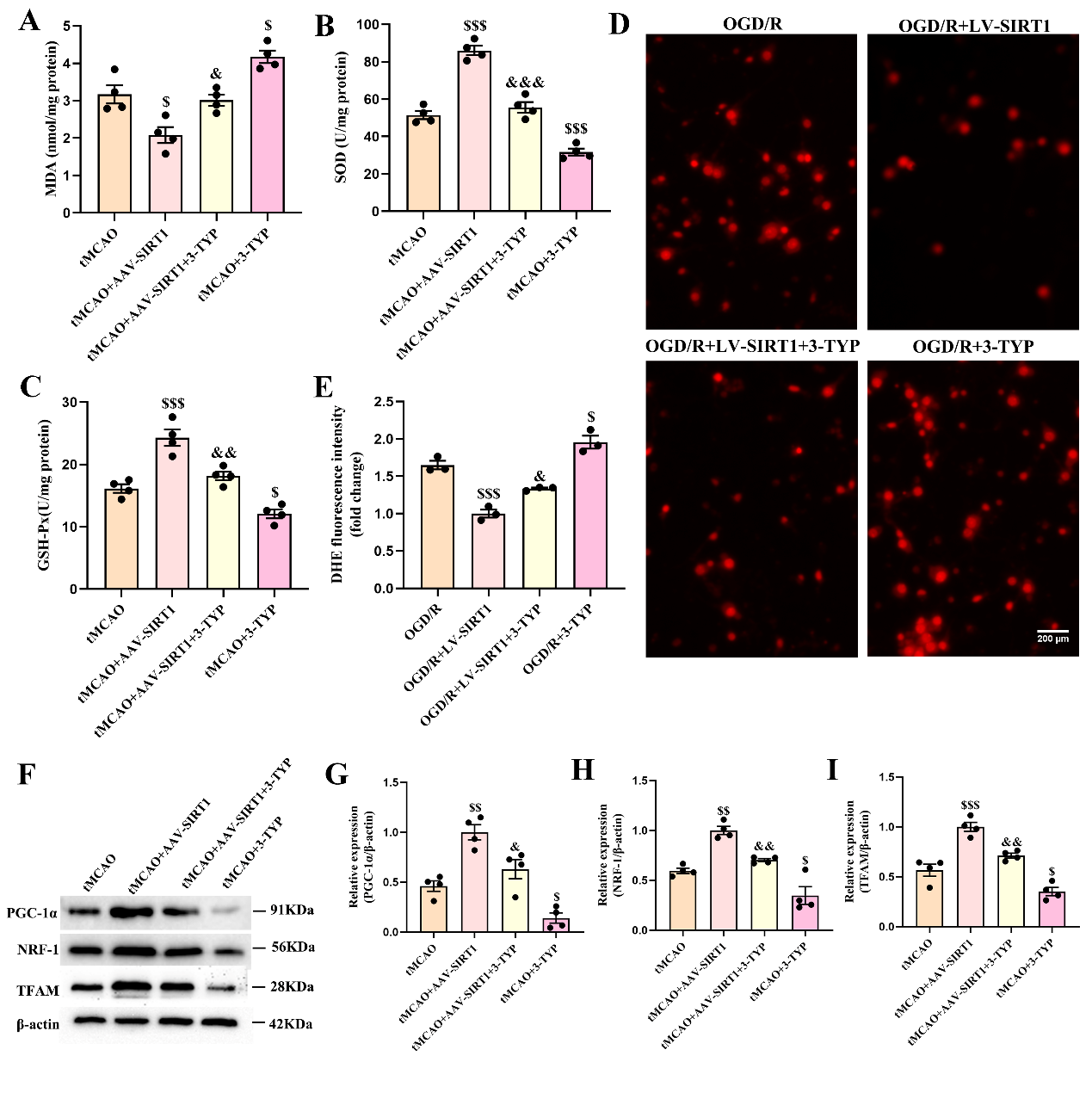
**

**Fig.S3. The alleviated oxidative effects and improved mitochondrial biogenesis after CI/R or OGD/R by SIRT1 via the enhancement of SIRT3 activity.** (A) The level of MDA, (B) the activity of SOD and (C) GSH-Px; n=4. (D) DHE representative fluorescence images and (E) Quantification of fluorescence intensity in neurons; n = 3. (F) Immunoblot analysis and (G) quantification of PGC-1α, (H) NRF-1 and (I) TFAM protein expression level; n = 4.

**4 SIRT1 retains** **mitochondrial integrity and alleviates the mitochondrial morphological damage after CI/R or OGD/R by enhancing SIRT3 activity**

We then examined the role of SIRT3 in the SIRT1-mediated protection of mitochondrial integrity. The analyses showed that the depolarization of the mitochondrial membrane potential was markedly aggravated in the neurons treated with OGD/R+3-TYP when compared with the OGD/R-exposed neurons. A similar aggravation was found in the neurons treated with OGD/R+LV-SIRT1+3-TYP when compared with the neurons treated with OGD/R+LV-SIRT1 (Fig. S4A-B). Moreover, the expressions of cytosolic Cyt c and nuclear AIF increased and the expression of mitochondrial Cyt c decreased in the tMCAO+3-TYP-treated rats when compared with the tMCAO-treated rats. Similar changes were observed in the rats treated with OGD/R+AAV-SIRT1+3-TYP when compared with the OGD/R+AAV-SIRT1-treated rats (Fig. S4C-F). These results suggest that SIRT1 retains mitochondrial integrity after CI/R or OGD/R, which can be partially abolished by 3-TYP.

Next, we investigated whether SIRT3 participates in the SIRT1-mediated protection of mitochondrial morphology after CI/R. The analyses showed that the length of mitochondria and the number of mitochondrial cristae decreased in the tMCAO+3-TYP-treated rats when compared with the tMCAO-treated rats. Similar changes were found in the rats treated with tMCAO+AAV-SIRT1+3-TYP when in comparison with the tMCAO+AAV-SIRT1-treated rats (Fig. S4G-I). These results confirm that SIRT1 alleviates the mitochondrial morphological damage after CI/R, which can be partially abolished by 3-TYP.


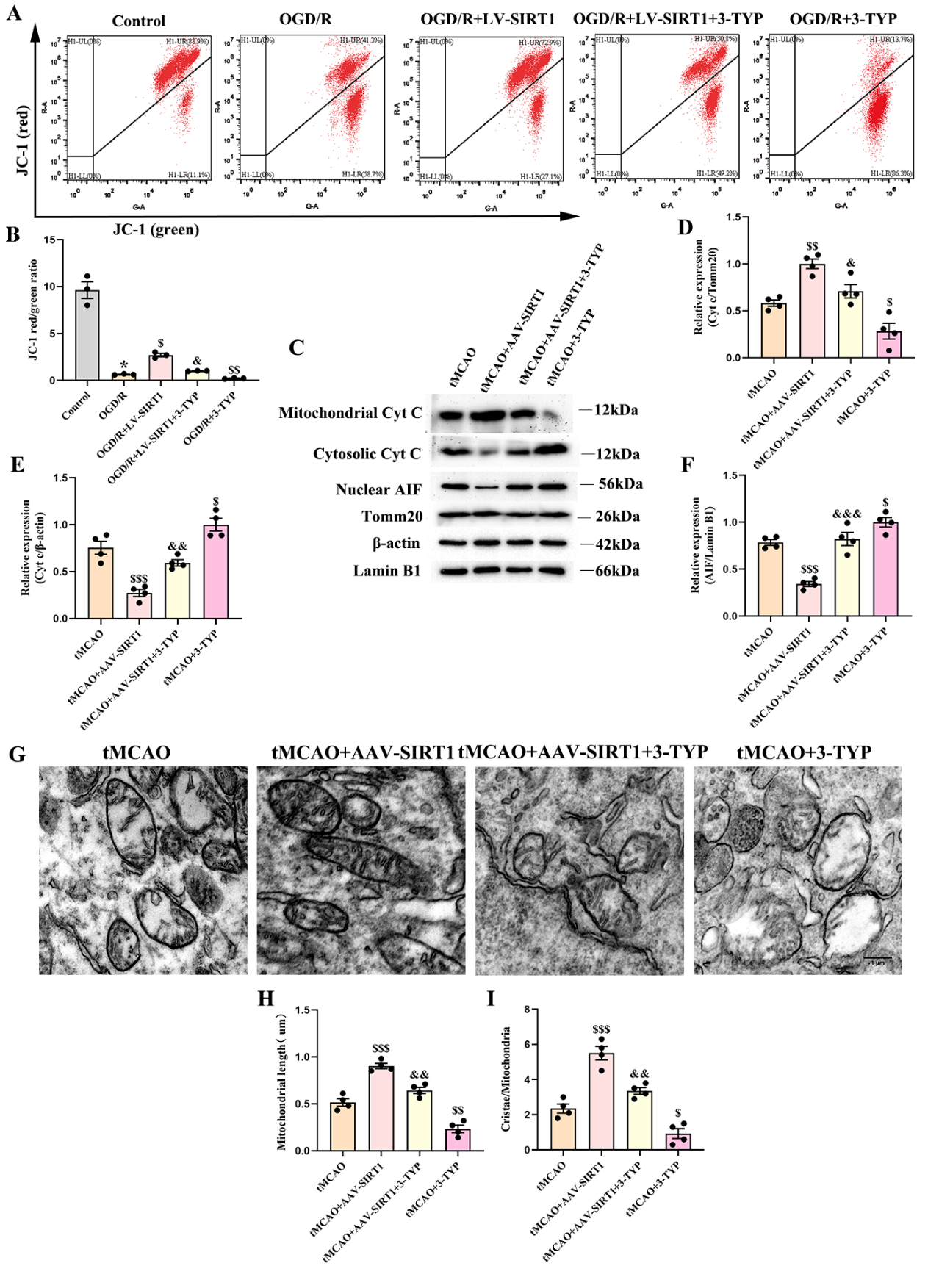


**Fig.S4. The retained mitochondrial integrity and alleviated mitochondrial morphological damage after CI/R or OGD/R by SIRT1 via the enhancement of SIRT3 activity.** (A) JC-1 expression analyzed by flow cytometric and (B) quantification of JC-1 expression in neurons; n =3. (C) Immunoblot analysis and (D) quantification of the mitochondrial Cyt c level, (E) the cytosol Cyt c levels, and (F) the nuclear AIF level; n = 4. (G) Representative TEM pictures. Scale bar: 1 μm. (H) Mitochondrial length; 50–60 mitochondria per experiment. (I) Mitochondrial cristae number; 50–60 mitochondria per experiment.

**5 SIRT1 improves mitochondrial respiratory function after OGD/R by enhancing SIRT3 activity.**

Then, we determined whether SIRT3 participates in the SIRT1-mediated protection of mitochondrial respiratory function in the cortical neurons. Compared with the OGD/R-exposed neurons, neurons treated with 3-TYP showed a decrease in basal respiration, spare respiration, maximal respiration, and ATP-linked respiration compared with the OGD/R-exposed neurons. The neurons treated with OGD/R+LV-SIRT1+3-TYP showed a similar decrease when compared with OGD/R+LV-SIRT1-treated neurons. However, no significant change in the proton leak-linked respiration was found in the above four groups (Fig. S5A-F). Besides, compared with the OGD/R exposed-neurons, the OGD/R+3-TYP -treated neurons showed a decrease in ATP contents. A similar decline was also evident in the neurons treated with OGD/R+LV-SIRT1+3-TYP when compared with the OGD/R+LV-SIRT1-treated neurons (Fig. S5G). Altogether, the above results suggest that SIRT1 improves mitochondrial respiratory function after OGD/R, which can be partially abolished by 3-TYP.


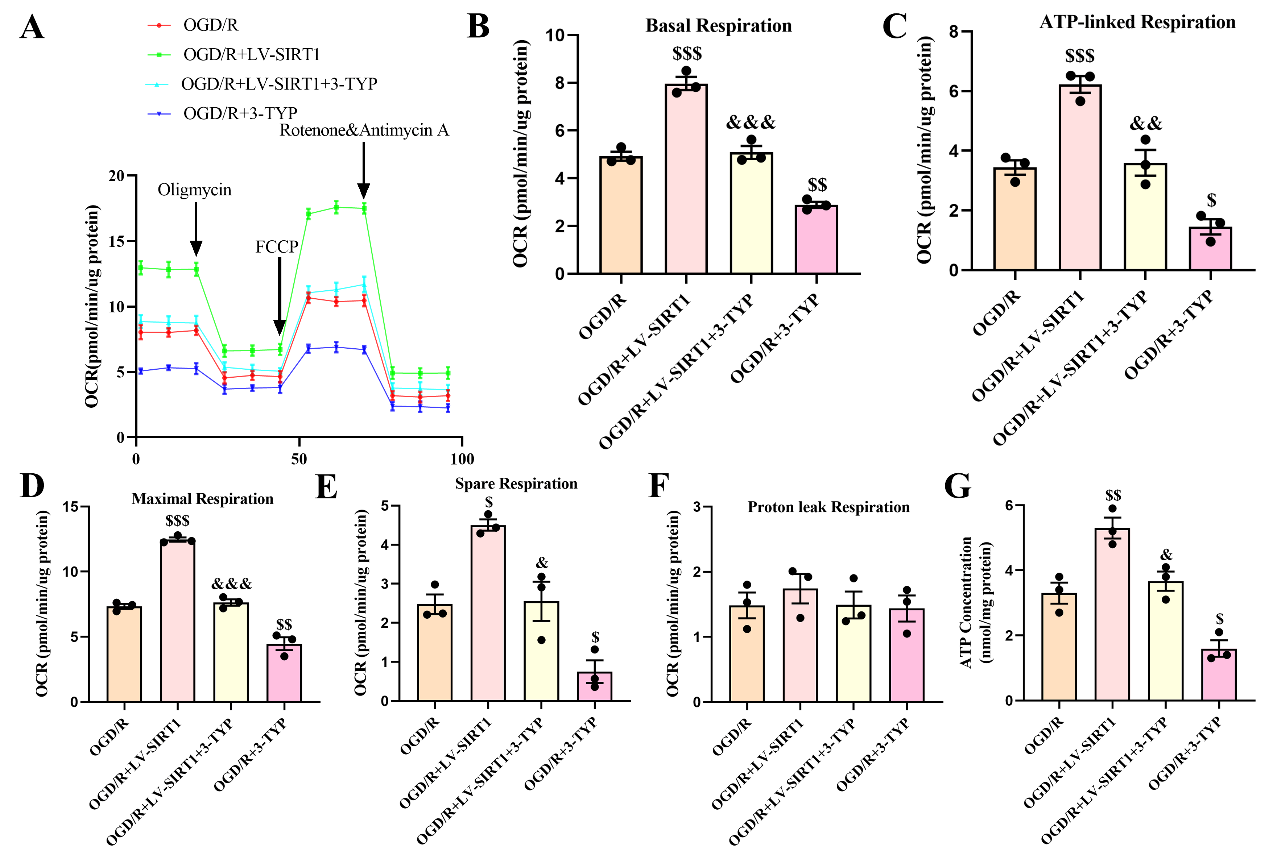


**Fig.S5. The improved mitochondrial respiratory function after OGD/R injury by SIRT1 via the enhancement of SIRT3 activity.** (A) Representative OCR profiles. (B) Basal respiration, (C) ATP-linked respiration, (D) maximal respiration, (E) spare respiration and (F) proton leak respiration; n =3. (G) Quantification of ATP levels in neurons; n = 3.

**Tables**

**Table S1 The characteristics of the primers used in this study**

| ***Number*** | ***Primer Name*** | ***Primer Sequence 5’→ 3’*** |
| --- | --- | --- |
| *1* | *Rat PGC-1α for* | *5’-CGCTGCTCTTGAGAATGGATAT-3’* |
| *2* | *Rat PGC-1α rev* | *5’-GTCATACTTGCTCTTGGTGGAA-3’* |
| *3* | *Rat SIRT1 for* | *5’-GACGCCTTATCCTCTAGTTCCT-3’* |
| *4* | *Rat SIRT1 rev* | *5’-CAGCATCATCTTCCAAGCCATT-3’* |
| *5* | *Rat TFAM for* | *5’-AATGTGGGGCGTGCTAAGAAC-3’* |
| *6* | *Rat TFAM rev* | *5’-ACAGATAAGGCTGACAGGCGAG-3’* |
| *7* | *Rat NRF-1 for* | *5’-ATGGACCATCAGCAAAGCCG-3’* |
| *8* | *Rat NRF-1 rev* | *5’-CAACGTAAGCTCTGCCTGGT-3’* |
| *9* | *Rat GAPDH for* | *5’-CGCTGCTCTTGAGAATGGATAT-3’* |
| *10* | *Rat GAPDH rev* | *5’-GTCATACTTGCTCTTGGTGGAA-3’* |
| *11* | cytochrome b *for* | *5′-GTTCGCAGTCATAGCCACAGCA-3′* |
| *12* | *cytochrome b rev* | *5′- AACGATTGCTAGGGCCGCGAT-3′* |
| *13* | *RPL13A for* | *5′-CTCAAGGTCGTGCGTGCGTCTG-3′* |
| *14* | *RPL13A rev* | *5′-TGGCTTTCTCTTTCCTCTTCTC-3* |

**Table S2 The primary and secondary antibodies used in Western blotting**

| ***Name*** | ***Cat#*** | ***Percentage*** | ***Company*** |
| --- | --- | --- | --- |
| *rabbit anti-Lamin B1* | *ab16048* | *1:5000* | *Abcam* |
| *rabbit anti-SIRT1* | *ab189494* | *1:1000* | *Abcam* |
| *mouse anti-Tomm20* | *ab56783* | *1:500* | *Abcam* |
| *anti-acetylated lysine* | *9441* | *1:1000* | *Cell Signaling Technology* |
| *rabbit anti-Bcl-2* | *ab59348* | *1:1000* | *Abcam* |
| *mouse anti-PGC-1α* | *sc-518025* | *1:500* | *Santa Cruz Biotechnology* |
| *rabbit anti-Bax* | *ab32503* | *1:5000* | *Abcam* |
| *rabbit anti-AIF* | *ab32516* | *1:1000* | *Abcam* |
| *mouse anti-NRF-1* | *sc-365651* | *1:500* | *Santa Cruz* |
| *mouse anti-Caspase-3* | *66470-2-Ig* | *1:3000* | *Proteintech* |
| *mouse anti-SIRT3* | *sc-365175* | *1:1000* | *Santa Cruz Biotechnology* |
| *mouse anti-TFAM* | *sc-166965* | *1:500* | *Santa Cruz Biotechnology* |
| *rabbit anti-Cyt c* | *ab90529* | *1:1000* | *Abcam* |
| *rabbit anti-β-actin* | *BM3872* | *1:400* | *Boster* |
| *goat anti-rabbit IgG-HRP* | *ab97040* | *1:5000* | *Abcam* |
| *goat anti-mouse IgG-HRP* | *ab97080* | *1:5000* | *Abcam* |

**Table S3 The free energy distribution inthe Complex (kcal/mol)**


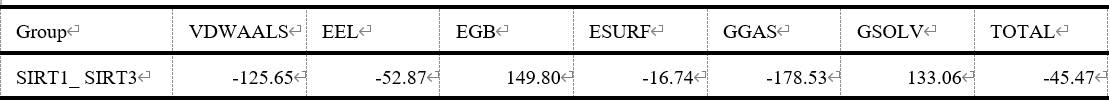


Note: VDWAALS: van der Waals energy; Eel: Electrostatic energy; EGB: Polar solvation energy; ESURF: Non-polar solvation energy; GGAS: Total gas phase free energy; GSOLV: Total solvation free energy.

| ***Name*** | ***Cat#*** | ***Percentage*** | ***Company*** |
| --- | --- | --- | --- |
| *rabbit anti-SIRT1* | *ab189494* | *1:1000* | *Abcam* |
| *mouse anti-SIRT3* | *sc-365175* | *1:100* | *Santa Cruz Biotechnology* |
| *mouse anti-neuron* | *ab104224* | *1:200* | *Abcam* |
| *rabbit anti-Cyt c* | *ab90529* | *1:1000* | *Abcam* |
| *Alexa Fluor 594 Donkey anti-Mouse secondary antibody* | *A21203* | *1:400* | *Thermo Fisher Scientific* |
| *Alexa Fluor 488 Donkey anti-Rabbit secondary antibody* | *A-21206* | *1:1000* | *Thermo Fisher Scientific* |

**Table S4 The primary and secondary antibodies used in Immunofluorescent staining**

**References**

Al Rahim, M, Thatipamula, S, Pasinetti, G M, & Hossain, M A. (2021). Neuronal Pentraxin 1 Promotes Hypoxic-Ischemic Neuronal Injury by Impairing Mitochondrial Biogenesis via Interactions With Active Bax[6A7] and Mitochondrial Hexokinase II. *ASN Neuro, 13*, 17590914211012888. doi:10.1177/17590914211012888

Che, Q, Wang, W, Duan, P, Fang, F, Liu, C, Zhou, T, . . . Zhao, K. (2019). Downregulation of miR-322 promotes apoptosis of GC-2 cell by targeting Ddx3x. *Reprod Biol Endocrinol, 17*(1), 63. doi:10.1186/s12958-019-0506-7

Pérez-Mato, M, Iglesias-Rey, R, Vieites-Prado, A, Dopico-López, A, Argibay, B, Fernández-Susavila, H, . . . Campos, F. (2019). Blood glutamate EAAT(2)-cell grabbing therapy in cerebral ischemia. *EBioMedicine, 39*, 118-131. doi:10.1016/j.ebiom.2018.11.024

Xu, X Y, Fang, Q, Huang, W, Li, B C, Zhou, X H, Zhou, Z Y, & Li, J. (2020). Effect of Electroacupuncture on Neurological Deficit and Activity of Clock and Bmal1 in Cerebral Ischemic Rats. *Curr Med Sci, 40*(6), 1128-1136. doi:10.1007/s11596-020-2295-9

Zhang, X, Wei, M, Fan, J, Yan, W, Zha, X, Song, H, . . . Wang, W. (2021). Ischemia-induced upregulation of autophagy preludes dysfunctional lysosomal storage and associated synaptic impairments in neurons. *Autophagy, 17*(6), 1519-1542. doi:10.1080/15548627.2020.1840796
