# Supplementary figures and images for: SIRT1 restores mitochondrial structure and function in rats by activating SIRT3 after cerebral ischemia/reperfusion injury
Source: Cell Biol Toxicol. 2024 May 20;40(1):31. doi: 10.1007/s10565-024-09869-2 (PMC11106166; doi:10.1007/s10565-024-09869-2)

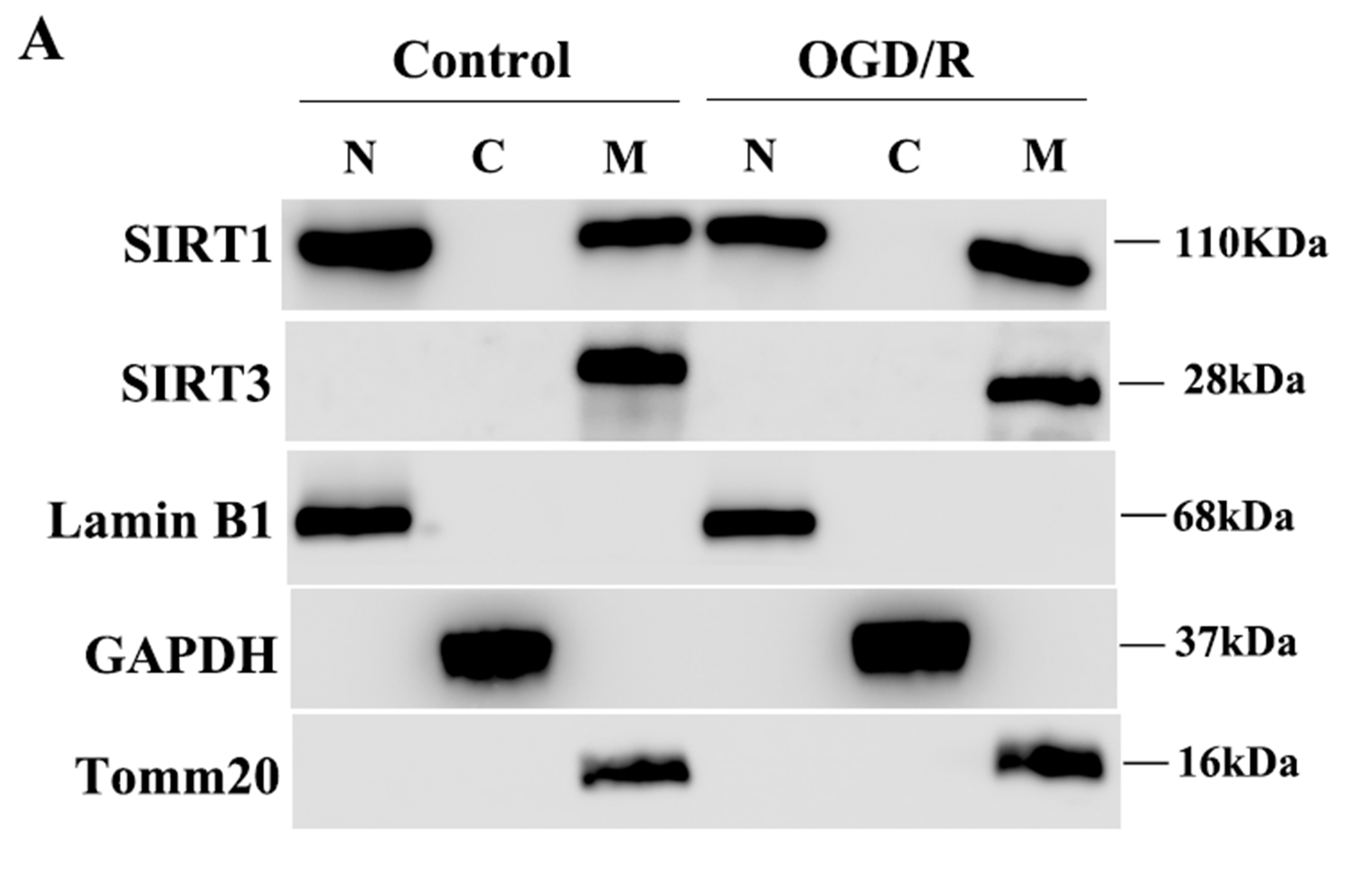

Supplement: Supplementary file 2 — (PNG 561 kb) [file 10565_2024_9869_Fig7_ESM.png]

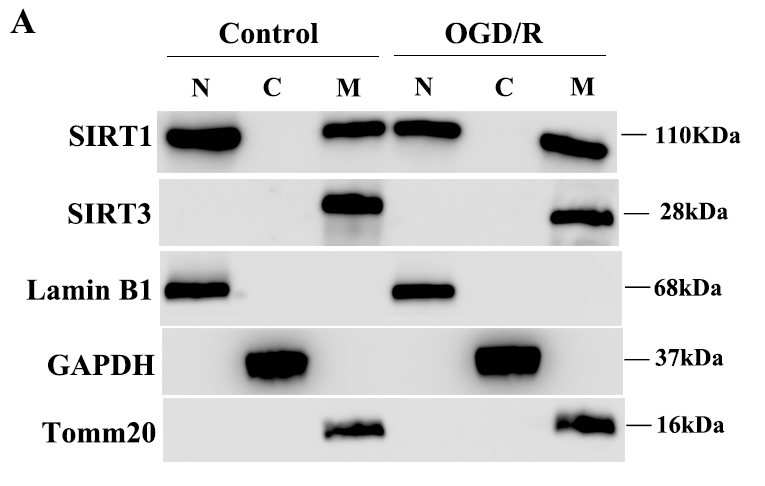

Supplement: Supplementary file 3 — (TIF 1.09 MB) [file 10565_2024_9869_MOESM2_ESM.tif]
